# Hybrid and Rogue Kinases Encoded in the Genomes of Model Eukaryotes

**DOI:** 10.1371/journal.pone.0107956

**Published:** 2014-09-25

**Authors:** Ramaswamy Rakshambikai, Mutharasu Gnanavel, Narayanaswamy Srinivasan

**Affiliations:** Molecular Biophysics Unit, Indian Institute of Science, Bangalore, India; University of Oulu, Finland

## Abstract

The highly modular nature of protein kinases generates diverse functional roles mediated by evolutionary events such as domain recombination, insertion and deletion of domains. Usually domain architecture of a kinase is related to the subfamily to which the kinase catalytic domain belongs. However outlier kinases with unusual domain architectures serve in the expansion of the functional space of the protein kinase family. For example, Src kinases are made-up of SH2 and SH3 domains in addition to the kinase catalytic domain. A kinase which lacks these two domains but retains sequence characteristics within the kinase catalytic domain is an outlier that is likely to have modes of regulation different from classical src kinases. This study defines two types of outlier kinases: hybrids and rogues depending on the nature of domain recombination. Hybrid kinases are those where the catalytic kinase domain belongs to a kinase subfamily but the domain architecture is typical of another kinase subfamily. Rogue kinases are those with kinase catalytic domain characteristic of a kinase subfamily but the domain architecture is typical of neither that subfamily nor any other kinase subfamily. This report provides a consolidated set of such hybrid and rogue kinases gleaned from six eukaryotic genomes–*S.cerevisiae, D. melanogaster, C.elegans, M.musculus, T.rubripes* and *H.sapiens*–and discusses their functions. The presence of such kinases necessitates a revisiting of the classification scheme of the protein kinase family using full length sequences apart from classical classification using solely the sequences of kinase catalytic domains. The study of these kinases provides a good insight in engineering signalling pathways for a desired output. Lastly, identification of hybrids and rogues in pathogenic protozoa such as *P.falciparum* sheds light on possible strategies in host-pathogen interactions.

## Introduction

Living cells constantly respond to both internal and external stimuli with the help of signalling systems. As the complexity of the organism increases, the complexity of signalling systems also increases [1], [2], [3]. Complexity may be manifested by the introduction of new molecular players or inter-molecular interactions that constitute a network. Enzymes involved in signalling process are often multi-modular in nature and have other domains in addition to the core catalytic domain that facilitate interactions with other elements in the signalling pathway. Moreover, the domains participating in signalling pathways have diverse functions. Hence, various permutations and combinations of different modules or domains of the signalling proteins lead to the evolution of complex networks of communicating modules [4], [5].

Many of the signalling domains function in the process of cellular localization, provide interacting partners and regulate the activity of the protein [6], [7], [8], [9], [10], [11], [12], [13], [14]. They also aid in spatio-temporal separation of proteins and thus prevent/facilitate cross talk, which is important in signalling systems. Domain recombination of signalling proteins therefore generates varieties in overall functions, which are then elected on the basis of requirement and specificity. Earlier studies have indicated that new features in molecular wiring are achieved by different combinations of already existing domains rather than recruiting new domain families [4]. However, the types of domain architecture seen in higher eukaryotes are more complex than those present in invertebrates. Therefore, mix and match of domain families seems to be the mechanism for the evolution of complex signalling networks [5], [15], [16], [17], [18], .

Protein kinases are a group of enzymes that play important roles in almost all signalling pathways. In this work, we consider Ser/Thr and Tyr kinases only. About 280 different subfamilies of protein kinases have been identified so far, and these are involved in regulating different parts of signalling pathways in various organisms [20]. The catalytic kinase domain family is highly promiscuous and is reported to be seen in ∼4500 different domain architectures [21]. In addition, there is a characteristic domain architecture for every subfamily of a kinase. Therefore, from the knowledge of subfamily, in principle, one might generate an expectation of domains which are tethered to the kinase catalytic domain and vice-versa [22]. For example, Src kinases are associated with SH2 and SH3 domains in addition to the kinase catalytic domain that help in its interactions and hence transmitting the signal within the cell [23], [24].

**Figure 1:**
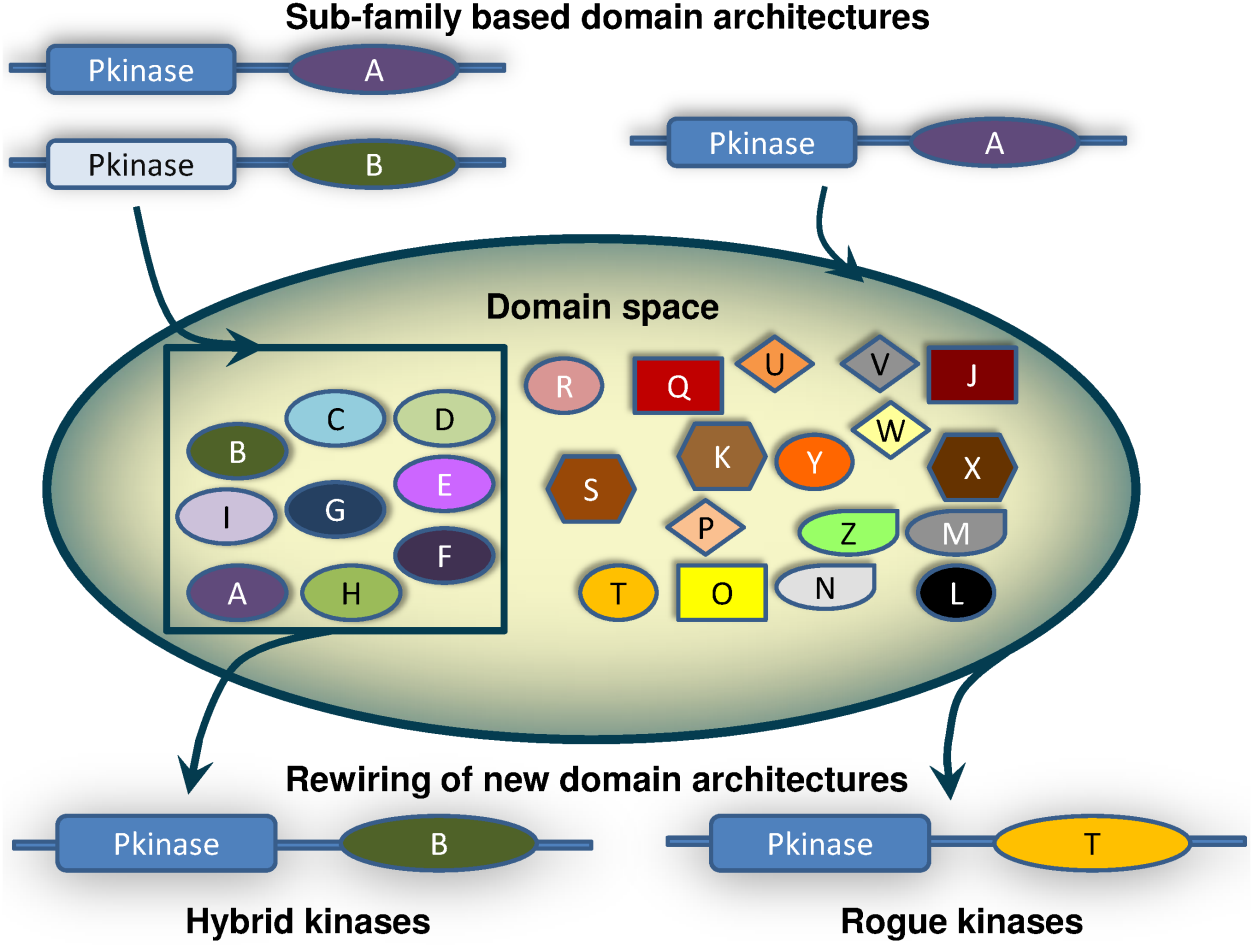
Evolution of domain architectures in kinases. The domain family space is represented as alphabets from A to Z. For the purpose of this Figure the kinase domain family is considered tethered to domain families A–I (inner pool), which are kinase sub-family specific, while J–Z (outer pool) are usually not observed tethered to kinase domain family. Shuffling of domains within the kinase domain family across sub-families leads to the birth of hybrid kinases. Recruitment of altogether new domain architectures from the outer pool leads to the birth of rogue kinases.

However, in nature, sometimes such subfamily-characteristic domain architectures may not be strictly followed [25]. With this feature in mind, the concept of “Hybrid” and “Rogue” kinases has been introduced. “Hybrid” kinases are those where the non-kinase domains and their sequential order are a characteristic feature of a kinase subfamily while the sequence patterns in kinase catalytic domains shows characteristic features of a different kinase subfamily. Therefore, these kinases show hybrid or chimeric properties with respect to their function. For example, an STE11 kinase, which is usually a single domain protein with only a single kinase domain in many organisms, is tethered to a Myosin_TH1 domain in *T. rubripes*. Therefore, this kinase is localized to the membrane due to the property of the domain associated with the STE11 kinase catalytic domain. Rogue kinases are those where the domain architecture of the kinase is not usually observed among currently known Ser/Thr/Tyr kinases. For example, the association of the DAXX domain with the TTBK subfamily of kinases indicates potential association of this kinase with transcriptional machinery. Such domain combinations may determine some of the properties of the protein and also introduce cross-talks in the pathway, thereby leading to more complex networks [17], [26]. These domain combinations may also aid in the adaptation of the organism to its respective surroundings. A pictorial representation of hybrid and rogue kinases is illustrated in Figure 1.

Kinases are classified into their respective subfamilies by means of clustering of sequences of solely kinase catalytic domains [27]. An inherent assumption here is that once the amino acid sequence of the kinase catalytic domain suggests a subfamily of the kinase, the associated domains, if any, will be characteristic of that subfamily of the kinase. The main point of this paper is that while this assumption is consistent with the classification of most kinases, it is inconsistent with the classification of many kinases. Due to such hybrid or rogue characteristics, these kinases may be grouped into new subfamilies, which are emergent subfamilies making them as new off-springs, in the evolution of multi-domain kinases, mediating cross-talks between signalling pathways or facilitating rewiring in an interaction network. A method developed earlier in our group, ClaP, has been shown to classify multi-domain proteins by considering the entire sequence with the complete domain intact, into subfamilies [28], [29]. This method has been extended in this study to identify “Hybrid and Rogue” kinases and validate their status as emergent new subfamilies of protein kinases.

Protozoans are known to exhibit various non-canonical features in their protein sequences [30], [31]. Therefore, later in this paper, a protozoan (*Plasmodium falciparum*) has been probed for existence of such sequences with hybrid or rogue features to probe if this feature is specific to higher eukaryotes alone or is also present in eukaryotic pathogens that attack them.

## Results and Discussion

Sequences of protein kinases from six model organisms (*H. sapiens, S. cerevisiae, M. musculus, T. rubripes, C. elegans,* and *D. melanogaster*) have been recognized by remote homology detection protocols adopted in previous publications from this laboratory [30], [32], [33]. Briefly, sequences containing kinase-like domains have been identified using RPS-BLAST [34] and the hmmscan [35] method, which identify kinases on the basis of their similarity to profiles of well known subfamilies of kinases: the details have been elaborated in the Methods section. Only those kinase-like sequences with the catalytic Asp conserved have been considered for further analysis, since the absence of the critical Asp residue does not guarantee kinase function. For an identified kinase, a subfamily is tentatively assigned if the sequence identity between the catalytic kinase domain and the catalytic domains of the members of the subfamily is greater than 30%.

It is well known that each kinase subfamily has a canonical domain architecture that determines the interactions, localization and overall function of the kinase. The 1498 sequences considered for the analysis have been classified into 91 different subfamilies on the basis of the sequences of their kinase catalytic domains. The canonical domain architectures characteristic for each of the 91 subfamilies of protein kinases considered is well known [27]. This information has been provided in Table S1 along with references supporting the information. Out of the 1498 kinases considered from 6 organisms, 1406 sequences have canonical domain architectures characteristic of their subfamily assigned by considering the amino acid sequence of the catalytic kinase domain only. However, 92 sequences have unusual domain architectures characteristic of either hybrid or rogue kinases. Out of the 92 kinases, only 18 cases show an altogether new recombination of domains for the kinase subfamily and are referred to as “Rogues”. Hybrid kinases are those which show similarity to one kinase-subfamily when only the catalytic domain sequence is considered and show characteristic domain architecture features of another sub-family of kinases. The complete list of hybrid and rogue kinases has been provided in Table S2. There is a higher number of hybrid kinases (74).

### Hybrid kinases

Among the hybrids, two kinds are generally observed. The first category of hybrid kinases is single domain kinases, but their classified subfamilies are typical of multi-domain kinases with specific domain architectures. For example, a classical PDGFR kinase, which is a receptor tyrosine kinase, is associated with Ig-like domains in the extracellular region and a membrane spanning region [36]. However, if a kinase classified as PDGFR is a single (kinase) domain protein, then it is annotated as a hybrid. The second kind of hybrid kinases correspond to multi-domain kinases with a catalytic kinase domain, and the domain architecture is different from the characteristic architecture of the classified subfamily.

#### Single domain hybrids

**Figure 2:**
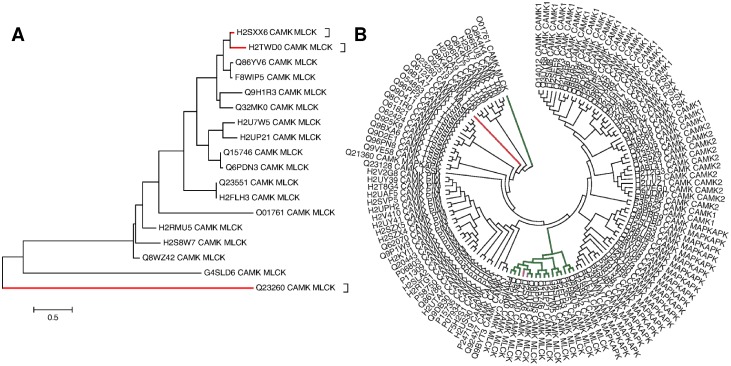
Phylogenetic relationships among the MLCK and CAMK subfamilies obtained using ClaP method. A) Dendrogram showing clustering of MLCK subfamily sequences in the dataset of six eukaryotes. Hybrid MLCKs are highlighted in red. B) Dendrogram showing clustering of CAMK sequences in the dataset. Classical MLCKs are indicated in green, Hybrid MLCKs are indicated in magenta (closely related to classical MLCKs) and red (distantly related to classical MLCKs). Scale bars indicate evolutionary distances as number of amino acid substitutions per site.

**Figure 3:**
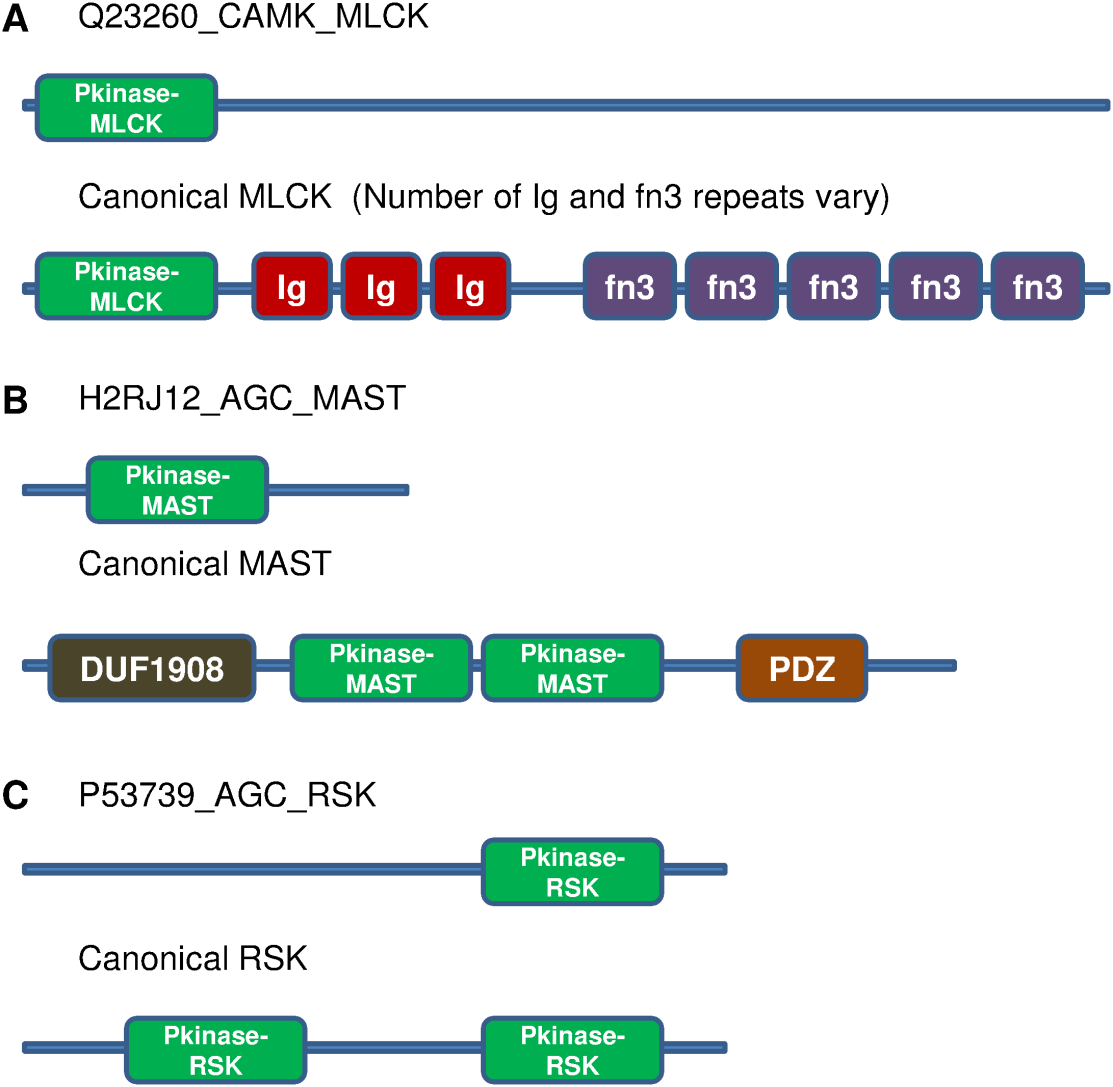
Representative examples of single kinase domain hybrids (top panel in each of A, B and C) and canonical domain architectures for the respective kinase subfamilies (bottom panel in each of A, B, and C). Sequence identities between the kinase domains of hybrid and canonical kinases are A) 83%, B) 89%, C) 47%.

Some of the kinase subfamilies such as PKA, CK1 and MAPK comprise single domain proteins with only the kinase catalytic domain. Some of these form higher order oligomers. However, if a kinase catalytic domain belonging to a kinase subfamily, which corresponds usually to multi-domain proteins, occurs as a single domain protein, it is said to be hybrid in nature. Here, the feature of being a single kinase domain protein is a characteristic of another subfamily corresponding to a multi-domain kinase. Thirty three of the seventy four hybrid kinases belong to this category, where the sequence correspond to a single domain protein corresponding to the kinase catalytic domain. Many of the sequences contain long un-assigned regions without being assigned to any domain family. Under strict norms these are unlikely to be single domain sequences. This section what is referred to as single domain hybrids mean that they contain a single assigned domain in the sequence. These sequences have been derived from well annotated genomes with high quality genome sequence data and, therefore, are unlikely to be truncated sequences. Therefore, these sequences were further explored to understand the similarities within the kinase domain. Maximum likelihood trees were generated using MEGA 5 for the subfamilies comprising such single domain kinases along with the kinase domains of the same subfamily but occurring as multi-domain kinases. By and large, these sequences do not vary much between kinase domains of single domain kinases and kinase domains of multi-domain kinases in the same subfamily (Figure S1). Sequence diversity could be noted in hybrids from PDGFR, MLCK and FAK, where there are changes within the kinase catalytic region, as reflected by these hybrids branching out as outliers in the dendrograms. The single domain hybrids are indicated in red in the case of the MLCK subfamily (Figure 2A), wherein the hybrid is clearly shown to be an outlier. The full length sequence of this protein was further compared with full length sequences of other subfamilies in the CAMK group, which traditionally consists of only the kinase catalytic domain. The maximum likelihood tree is represented as a dendrogram in Figure 2B. As observed in the earlier tree, only one of the hybrids is an outlier to the MLCK subfamily, indicated in red, which occurs as an outlier between Trbl and TSSK subfamilies. Therefore, although the kinase catalytic domain shows 43% sequence identity to the MLCK subfamily, the overall sequence indicates that the protein is likely to have a hybrid function. The canonical domain architecture of this subfamily along with that of the single domain hybrid are shown in Figure 3A. Similar domain architecture representations for other single domain hybrid kinases that are discussed below are shown in Figures 3B and 3C.

Laboratory experiment-based characterization of some of these hybrids has been reported in literature and are discussed here. The sequence H2RJ12 has homologues in drosophila, known as the Greatwall kinase, and MASTL in humans. These homologues have a single kinase domain (Figure 3B). Such occurrences in the MAST subfamily are rare and therefore, these sequences are also referred to as MAST-like and are much longer in length [37]. They act as phosphatase inhibitors and are different from classical MAST, which usually associate with phosphatases via PDZ domains. Another example is that of Flippase kinase in yeast (P53739), which is a single kinase domain belonging to RSK subfamily (Figure 3C). The RSK subfamily is usually characterized by the presence of two tandem kinase domains, where one of the kinase domains has a regulatory role in activating the other. However, this kinase has a single kinase domain and is activated by another kinase Ypk1 [38]. This again displays hybrid nature at the level of regulation of the kinase activity. Such single domain kinases in lower eukaryotes describe functions that have been segregated in earlier lineages and later integrated in higher eukaryotes by acquiring new domains that incorporate both functions.

#### Multi-domain hybrid kinases

This class of hybrid kinases contain a kinase catalytic domain which could be associated with a known sub-family of kinases solely on the basis of sequence features of the catalytic kinase domain. However, the domain architecture represents the prototype of another subfamily of kinases. Therefore these are likely to vary in their overall function and in terms of localization or regulation or interaction with other proteins depending on the functions of the domain and the domain architecture. Table S2 lists forty one cases identified as multi-domain hybrid kinases in this study.

**Figure 4:**
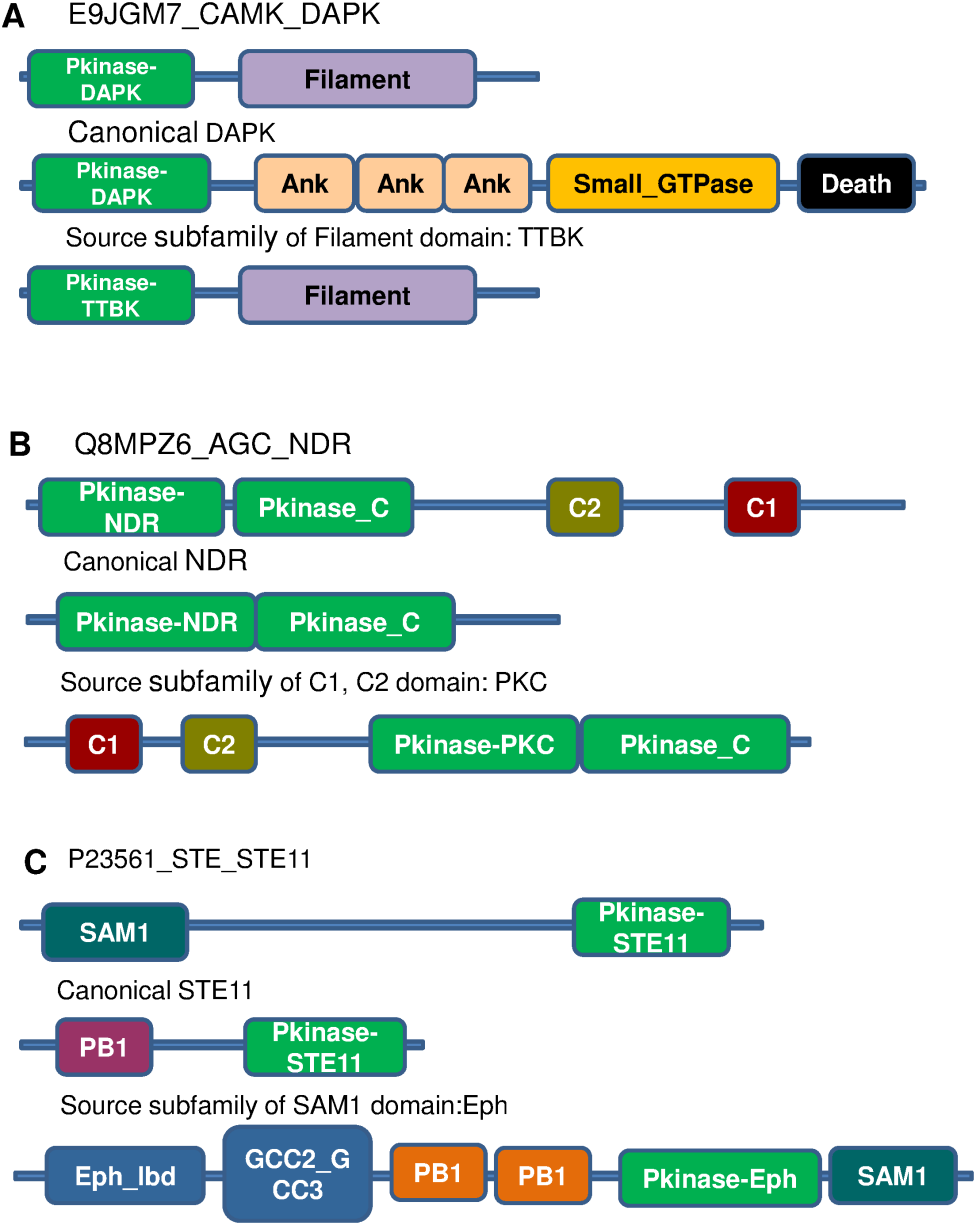
Domain architectures of three multi-domain hybrid kinases (A, B, C). Canonical domain architectures of classified subfamily and source subfamily are shown. Sequence identity between kinase domains of hybrid and canonical members of classified subfamily, A) 83%, B) 84%, C) 49%.

Hybrid nature of some of these kinases is discussed below. Their domain architectures and that of their canonical subfamily are shown in Figure 4.

One of the examples with localization likely to be unusual is the case of a DAPK associated with a filament domain (E9JGM7) (Figure 4A). The DAPK (Death Associated Protein Kinase), as the name suggests, has a major role in initiating apoptosis by both caspase-dependent and independent pathways. DAPK is usually localized in the cytoplasmic region, where the kinase is responsible for the phosphorylation of various proteins interacting with the pro-apoptotic protein, Bcl-2, thereby inducing apoptosis in a phosphorylation dependent manner. The filament domain is usually responsible for localization to the cytoskeletal/nuclear envelope region [39]. This particular hybrid kinase, which is a product of alternate splicing, is known as the ZIP kinase and has properties similar to those of canonical DAPK (sequence identity to canonical DAPK is 83%), in apoptosis; but by virtue of the presence of the filament domain, this kinase is localized to the membrane and is reported to be directly involved in membrane blebbing during the process of apoptosis [40].

The NDR kinase plays a role in regulating the MAPK pathways. An example of a hybrid is an NDR kinase associated with C1 and C2 domains (Q8MPZ6) (Figure 4B). Classical NDR kinase is a single domain kinase [41], which is usually regulated by phosphorylation. In PKC, regulation is brought about by binding to diacylglycerol and Ca^2+^ ions at the C2 and C1 domains, respectively [42]. Hybrid kinases in which NDR kinase, C1 and C2 domains are combined has been identified in other organisms as well. Such kinases are likely to display different modes of regulation compared to the classical NDR kinases.

Certain domains mediate protein-protein interactions. One such example is the SAM domain which induces dimerization in the proteins containing them. This domain is specifically seen to be associated with the Eph receptor family [43] which helps in the dimerization of Eph receptors (Figure 4C). Thus, combination of such a domain with the kinase catalytic domain suggests an elegant mechanism for dimerization in certain kinases that are otherwise single domain kinases in monomeric form. This domain has been studied in the context of STE11, which is a MAPKKK in yeast (P23561) and mediates interaction with the adapter protein STE50 in yeast [44], where dimerization with the adapter protein enables interaction with other proteins in the pathway. Several homologues of various proteins in the MAPK pathway are present in the cell; each of these have different binding properties such as differential binding to adapters and scaffolds in order to prevent cross-talk and leaky activities. The presence of such hybrids enhances the specificity of the MAPK pathway in yeast [45].

### Rogue kinases

Domain recombination leading to multi-domain proteins is instrumental in the evolution of signalling pathways. Certain domain architectures are more commonly observed involving certain domain families. Although there are such preferences, deviations do occur where domains are recruited in such a way so as to result in proteins with uncommon domain combinations leading to new functional features. From the dataset, 18 such rogue kinases have been identified, and they impart a wide range of functions to protein kinases.

**Figure 5:**
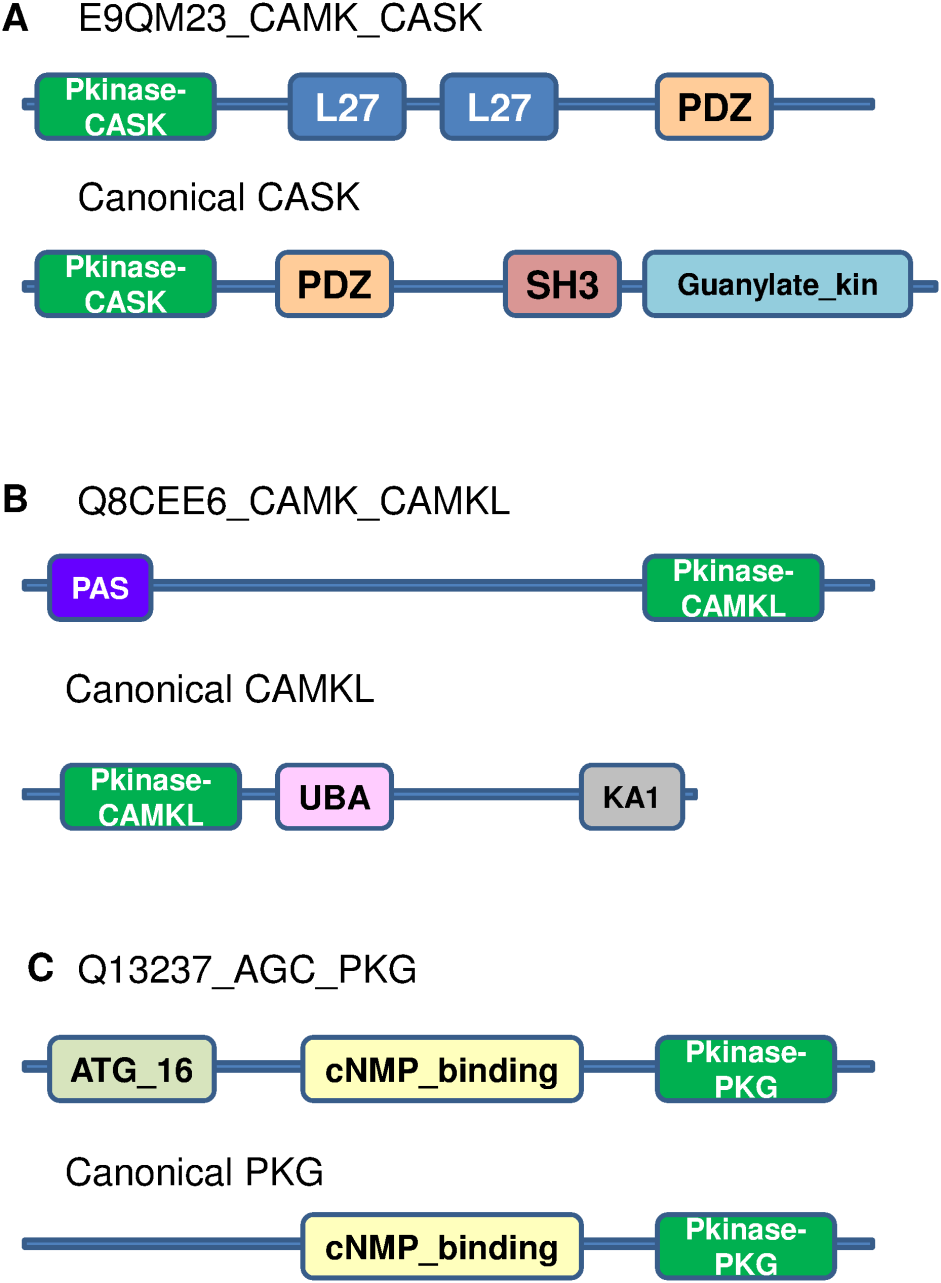
Domain architectures of three rogue kinases. Domain architectures of corresponding classical kinase subfamily are also shown in each panel. Sequence identities of kinase domain of rogue kinase with that of the canonical kinase of classified sub-family A) 47%, B) 37%, C) 97%.

The domain architectures of the rogue kinases identified in the current study are shown in Figure 5. The CASK is a multi-domain scaffolding kinase which has a role in synaptic trans-membrane protein anchoring and ion channel trafficking. The L27 domain is a protein interaction module that is present in many scaffold proteins with a role in cell polarity. Rogue kinase related to CASK (Figure 5A) is a variant associated with the L27 domain, which is known to interact with the N-terminal region of SAP97, resulting in lateral localization. SA97 mediates clustering of receptor molecules at the cell membrane. This rogue kinase was studied experimentally and shown to be well conserved in mammalian systems [46]. Therefore, the recruitment of L27 permits specific localization to the baso-lateral surface of the cell, where it serves as a scaffold for clustering membrane receptors.

Calcium/Calmodulin dependent kinases are of various types, some of which are involved in glucose metabolism by phosphorylation of Glycogen-synthase [47]. The PAS domain is seen in bacteria and fungi and is reported to be a modular sensor domain of the intracellular environment responding to changes in light, oxygen, redox states etc. The PAS domain associated kinase (Figure 5B) is another example of a rogue kinase. The PAS associated domain in mammalian systems also imparts a similar sensory role to the kinase implicated in maintaining glucose homeostasis and responding to hypoxia [48], [49], [50]. The sensory role of the PAS domain in integrated to the glucose metabolism role of the CAMK domain, thereby leading to a cross-talk between the stress related pathways and glucose metabolism.

Similarly, functions of such proteins could be extrapolated depending on the domains associated with the kinase domain and experimental studies that may indicate the rogue nature. An example of this is the Q13237 (Figure 5C), with the kinase catalytic domain associated with AGC group, and this kinase domain is tethered to ATG16 domain. AGC kinases play a major role in core intracellular pathways. PKG phosphorylates a number of proteins and is implicated in pathways regulating smooth muscle relaxation, platelet function, cell division and nucleic acid synthesis. The ATG16 domain is involved in autophagy. Experimental studies show the loss of this protein during immortalization. This indicates the recruitment of this protein in apoptotic pathways [51].

### Clustering of Hybrid and Rogue kinases

**Figure 6:**
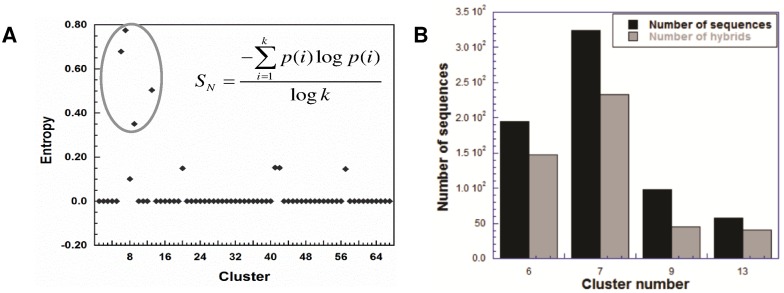
Graphs showing cluster analysis obtained upon hierarchical clustering of 1498 kinases. A) Entropy of various clusters, B) Proportion of hybrid/rogue kinases in clusters with high entropy.

The set of 92 outliers presented as hybrids and rogues in the sections above have been identified on the basis of comparison of domain architecture of the sequence with the cognate architectures of the classified subfamily. These hybrids/rogues are likely to be functionally different from the corresponding classical subfamily and hence need to be resolved and differentiated from them. In other words, these hybrid and rogue kinases are new emergent subfamilies that may be evolutionary offsprings of two subfamilies displaying hybrid function. The ClaP method developed earlier in this group [28] was used, and the tree has been further classified into clusters (Figure 6), as described in the Methods section. It has been well established by Bhaskara et. al [29] that this method clusters the sequences in concordance with the number of subfamilies. The entropy of each cluster gives an estimate of the subfamily variations within the cluster. The entropy for each of the clusters is provided in Table S3 and represented in Figure 6A. The 92 hybrids/rogues were then mapped in their respective cluster. It was observed that the clusters populated by hybrids and rogues have a high entropies, indicating that these clusters contain sequences from different sub-families. Further, clusters with high subfamily variation (entropy>0) were assessed (clusters showing group level entropy of zero were ignored). These clusters not only contain the 92 hybrids/rogues but also a large number of sequences with large inserts/overhangs (≥100 residues) at their N/C terminus without any recognized domains. Such sequences may also be considered as hybrids as they contain regions that are significantly diverged from the current domain families in Pfam database [21]. With the increase in the number of domain families, these regions may be assigned to domains.

Figure 6B compares the total number of sequences in clusters with high entropy (black) to the number of hybrids (grey) among these sequences. The concentration of hybrids and rogues only in certain clusters further validates the classification of these sequences into emergent sub-families.

### A note on hybrid and rogue kinases in *P. falciparum*

**Figure 7:**
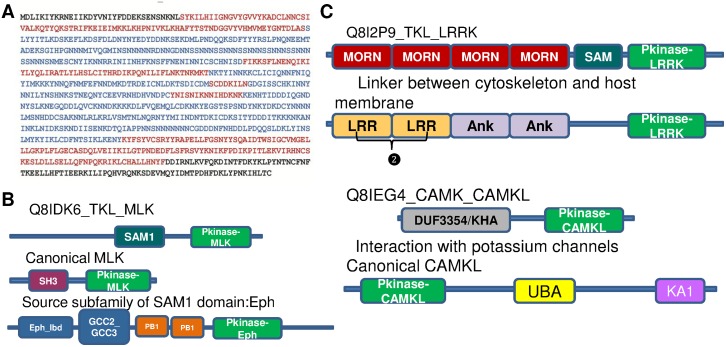
Features of *P.faciparum* sequences. A) *P.falciparum* MAP kinase showing kinase domain highlighted in red and *P.fal* specific inserts highlighted in blue. B) Domain architecture of hybrid kinase, canonical architectures of classified and source subfamily are shown C) Domain architecture of rogue kinases and canonical architectures of classified subfamily shown.

*P. falciparum* is an obligate parasite and shows a lot of variations in kinase distribution, with certain subfamilies being absent altogether like the MAP2K kinases and many members of the STE group [30], [31], [52], [53], [54]. In addition, certain subfamilies such as the CDPK are represented in fairly high numbers, which is usually a characteristic of plant genomes. These CDPK’s contain 2 or 4 EF-hand domains that are involved in Ca^2+^ binding and regulation. This protozoan organism not only shows variation in its distribution but also at the level of the sequence. Fifty-seven *P. falciparum* kinases were analyzed for their hybridnature. Forty-two of these kinases are either hybrids or rogues and these are listed in Table S4. Most of them are characterized by long N/C terminal overhangs or insertions within the kinase domain. The insertions within the kinase domain span across several residues and are marked by low complexity regions consisting of long stretches of polar groups like Asn and Gln. An example of this is shown in Figure 7A. The occurrence of such inserts in the *Plasmodium* genome is very well studied, although their evolutionary significance and function is largely debated. Structural studies on these proteins indicate the presence of zinc fingers in such regions [31]. However, an exhaustive study that would give an insight into parasite biology and the evolutionary importance of such inserts is warranted.

In addition to sequences containing such inserts, many kinases belong to the single kinase domain hybrid category that may be extrinsically regulated by the host proteins as well. Another hybrid is the MLK and Dicty4 subfamily kinase with the SAM domain tethered to it (Figure 7B), which is a characteristic of the Eph family implicated in hetero-dimerization. Such hybrids are well studied in yeast STE11 and are present in other genomes as well [45].

Two rogue kinases have been identified in the *Plasmodium* kinome (Figure 7C). One of them is that of the LRRK kinase, which is classically associated with LRR and Ank repeats. This kinase is associated with MORN repeats. These MORN repeats were first identified in parasite *Toxoplasma gondiis.* Variants of this architecture have been seen in several parasites, including *Leishmania* and *Trypanasoma* species. This protein has been reported to function as a linker between host membrane proteins and the cytoskeleton of the parasite [55]. Another rogue kinase in *P. faclciparum* is a CAMKL kinase tethered to a DUF3354 domain. This domain architecture again is represented in few parasites. This domain is annotated as the KHA domain in INTERPRO, which is the counterpart of the DUF3354 in Pfam. This protein is involved in the interaction of potassium channels in plants [56], [57], [58]. These rogue kinases represent a parasite specific architecture that may play a crucial role in interaction with the host proteins.

### Implications for rewiring/engineering signal transduction pathways

Tweaking with complex systems may result in unexpected signalling outcomes. Therefore, ideas from natural systems may be adopted to rewire certain signalling pathways to achieve desired outcomes. A review by Hohmann et al. [59] discusses in detail the design principles for rewiring signal transduction pathways. It also highlights the various lessons from previously engineered yeast cells specifically in the context of the MAPK pathway. Inspirations from the review and the results from this work could be used to explore further possibilities. A few examples of such possibilities have been discussed below.

New triggers for pathways: Pathways are usually triggered by the binding of ligands, which activate the receptor tyrosine kinase and subsequently, a whole array of signalling events. A rogue in this study where a PAS domain is tethered to a CAMKL kinase. A rewiring using the PAS domain and a receptor tyrosine kinase can be used as an effective replacement for conventional receptor kinases. The PAS domain could specifically add sensory functions so as to activate the pathway such as light, osmotic stress etc. in cultured cells.

Inducing programmed cell death: The ATG16 domain has a crucial role in triggering programmed cell death. Tethering of this domain to a protein kinase would help in activating apoptotic pathways. Activation of the chimeric kinases could be achieved by various mechanisms such as oligomerisation or phosphorylation or ligand binding. These chimeric proteins therefore function as an intermediary that facilitates cross-talks between two pre-existing pathways (first to activate this kinase and second to induce cell-death). Such chimeric proteins could be introduced into tumour cells to induce programmed cell death. The examples of hybrids and rogues discussed in this study could help in widening the prospects for designing more synthetic cell circuits.

## Conclusions

The protein kinase family is crucial in regulating important cellular pathways in the cell. Kinases are promiscuous in nature and occur with many associated domains that help in its localization, regulation and interaction with other proteins so as to relay the signal in a specific and time dependent manner. These kinases have been classified into groups and subfamilies that give an indication on the function based on specific motifs within the kinase catalytic domain. Although this has proven to be useful in a large number of cases, there exists a sub-population of kinases that have “inconsistencies” in the subfamily classification and associated domain combinations. We refer to them as hybrid and rogue kinases.

This study provides a consolidated list of such kinases from 6 eukaryotes and a eukaryotic pathogen. The AGC group is largely represented in the list of hybrid and rogue kinases identified. This is specifically interesting because the AGC kinase group comprises of proteins involved in core intracellular signalling and are subject to various modes of regulation including phosphorylation, binding to small molecules and forming higher order oligomers [60]. In addition, the overall number of rogues identified among the 88 cases is far lesser than that of hybrids. This provides an interesting insight into the recombination of domains. Previous interesting studies indicate that the various possibilities of domain recombination is domain family dependent; therefore, the tethering of domains outside the regular pool of tethered domains to a specific domain is a rather rare phenomenon [15], [16], which is evident in our study as well.

These hybrid kinases, due to their dual functional properties, may be eventually classified into separate subfamilies that constitute such outliers although their kinase catalytic domain shows significant similarity to one of the currently known subfamilies. The method presented to identify such novel and rare kinases on the basis of their local matching score identifies specific clusters that have high population of hybrid kinases, thereby re-iterating the fact that such kinases can be classified as a new subfamily.

Some of the domain architectures represented in these hybrids and rogues are more commonly noted across organisms while others are more organism-specific or may be referred to as orphan kinases. The objective of this study was to identify all deviant kinases. The deviant architectures seen only once so far have been marked as orphans in Table S2. Some of these kinases are experimentally studied and described to provide specific functional advantage to the organisms. In addition, the orphan status of some of these kinases is likely to change with sequencing of genomes of related organisms considered in this study providing further validation to their lineage specificity and the exact time of origin of such recruitments.

The presence of hybrid and rogue kinases indicates an elegant evolutionary mechanism that causes variations in the signal transduction pathways that are important for the adaptation of an organism especially in case of pathogens. Study of such hybrid kinases also provides an understanding for engineering signal transduction pathways for a desired output. Such a mechanism of domain recombination leading to evolution/rewiring of signal transduction pathways has been described in a review by Bhattacharya et al. [17]. Study of such domain architectures serves as a platform to construct synthetic cell circuits, which has a wide-range of bio-technological applications whose potential has been highlighted in a few earlier studies [15], [61], [62], [63], [64].

## Materials and Methods

### Identification of protein kinases

Protein kinase sequences encoded in the genomes of these organisms have been identified using a well-established protocol involving profile search methods adopted previously [65], [66], [67], [68]. Briefly, profiles for classical kinase subfamilies were built from those defined in http://kinase.com [68], [69], [70], [71]. Using an RPS-BLAST search [34] with an e-value cut-off of 10^−4^ on sequences greater than 200 residues (the length cut-off of 200 residues has been chosen since the kinase domain is about 200–300 residues long) in the genome, an initial set of kinase-like sequences were identified. These were then filtered out using a profile coverage criteria of ≥70% to weed out false positives. A sequence is assigned to a particular subfamily of Ser/Thr/Tyr kinase only if it shares at least 30% sequence identity with the profile of that subfamily.

### Identification of putative active kinases

The catalytic Asp is the most crucial residue for the kinase to be active since it mediates the phosphate transfer [72]. Therefore, the sequences in the dataset have been verified to have the catalytic Asp conserved. To do so, a multiple sequence alignment of the kinase catalytic domain region was performed using ClustalW [73]. The catalytic residue has been identified on the basis of the consensus pattern similar to HR**D**LKXX**N**. The most crucial residue is the Asp, which is usually identified by conservation of Asn four residues further. Substitutions in other residues (H, R, L, K) have been observed in certain sub-families which are likely to be still functional.

### Dataset of protein kinases

The datasets of protein kinase sequences have been compiled from six model organisms viz. 1. *Homo sapiens* 2. *Saccharomyces cerevisiae* 3. *Caenorhabditis elegans* 4. *Drosophila melanogaster* 5. *Takifugu rubripes* 6. *Mus musculus.* Only the sequences belonging to the well-characterized protein kinase subfamilies for which all level of function are well established were considered for this analysis. The outlier group also known as the “Other” group has been excluded from this analysis due to debated gross level function annotation for these subfamilies. The complete list of kinases identified from these 6 genomes and their sub-families obtained by considering solely the kinase catalytic domain are provided in the Table S5. A total of 1498 sequences, were used to identify “hybrid and rogue” kinases. Further, a set of 57 kinases from *Plasmodium falciparum* (Table S5) has also been analysed. With respect to the human kinome, we have used the latest dataset of human genome sequence and performed an extensive analysis of hybrid and rogue kinases encoded in the human genome (R. Rakshambikai, M. Gnanavel & N. Srinivasan, submitted for publication).

### Domain architecture assignment

Domains in the proteins used for the analysis have been assigned using the hmmscan program searched on the domain-wise hmm profiles from the Pfam v26 database [21] using an e-value cut-off of 0.01. In case of two domain assignments in the same region of the protein, the domain with longer span and better e-value has been selected.

### Identification of hybrid and rogue kinases

Totally 91 subfamilies across 7 groups were considered for the analysis. The characteristic domain architecture of each of the 91 subfamilies was compiled on the basis of thorough literature survey. Domain assignments for each of the 1498 sequences was made on the basis of HMMSCAN program [74] using Pfam-A HMM profiles provided in Table S5. The domain architectures of 1498 sequences, with kinase domain in each of these sequences corresponding to a well-known subfamily, were then compared with the domain architectures of appropriate subfamilies of kinases known from the literature. This comparative study enabled us to identify non-canonical domain architectures. Sequences with non-canonical domain architectures were classified into “Hybrid” and “Rogue” kinases depending upon combination of non-kinase domains with the kinase domain corresponding to a sub-family in the dataset of 1498 kinases.

### Clustering and generation of trees

Dendrograms for the single kinase hybrids were generated using the Maximum likelihood method as implemented in the MEGA5 [75] package using the JTT (Jones-Taylor-Thornton) model with uniform rates for every site. The initial tree is automatically generated using neighbour joining method. Maximum likelihood (ML) trees are then inferred by a heuristic method where the branches are swapped to optimize for trees, using the MEGA5 package, that give the highest ML value. The final tree is a result of several rounds of ML estimation that gives the tree optimized for most probable topology and branch length.

Full-length sequences of 1498 kinases were comparatively analyzed using the alignment-free method [28] to generate a dendrogram based on Local Matching Score (LMS) or ClaP method. The details of the method is described in greater detail by Martin et al. and Bhaskara et al. [28], [29]. Briefly, it scans five residues stretches between the two proteins and assigns a score considering only identical matches.
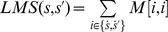
where 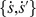 denotes the set of amino scids from the two proteins that are part of the 5 residue stretch and *M*[*i,i*] is the BLOSSUM62 substitution score. The scores are then normalised to give distance measures which ranges from 0 to 1. The distance matrices are used to obtain the trees. An indirect method has been employed to ascertain reliability to the tree since no direct bootstrapping methods are available for trees generated using alignment free methods. This has been discussed in Text S1.



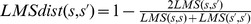



The dendrogram is parsed at 0.25 cut off to obtain clusters by hierarchical clustering using Wards method as employed in R package. The individual clusters give an estimate of the possible subfamilies that the dataset can be divided into. Since subfamily information based solely on the sequence of kinase catalytic domain is well known, the variation of subfamilies within each cluster was estimated as a function similar to the Shannon entropy score.
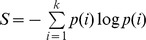
where, *i* is a given Hanks and Hunter subfamily, *k* is the total number of kinase subfamilies considered and *p(i)* is the fraction of sequences belonging to a subfamily *i* in a particular cluster. Details of the scoring schemes have been described in greater detail by Bhaskara et al. [29]. In short, the scores are normalised from 0 to 1 where 0 indicates completely pure clusters.

## Supporting Information

Figure S1 Maximum likelihood trees showing various subfamilies that contain hybrid/rogue kinases with the canonical cases in black and hybrids highlighted in red and rogues are highlighted in green. Scale bars indicate distances as number of amino acid substitutions per site. A) Eph, B) Focal adhesion kinase, C) Met, D) Ror, E) Fer, F) PKC, G) Src H) PDGFR I) MAST and J) NDR.
(PDF)

Table S1 Canonical domain architectures for each of the 91 subfamilies used in the study based on literature survey.
(DOCX)

Table S2 List of hybrid and rogue kinases from the six model eukaryotes *S. cerevisiae, C.elegans, D.melanogaster, T.rubripes, M.musmusculus, H.sapiens.*
(DOCX)

Table S3 Clustering of the 1498 sequences using full length alignment free method. Number of sequences, entropy score and subfamily variation for each cluster are also provided.
(DOCX)

Table S4 List of hybrid and rogue kinases from *P.falciparum.*
(DOCX)

Table S5 Domain architectures, subfamily information and the length of 1498 sequences from 6 eukaryotes and 57 sequences from *P.falciparum.*
(XLSX)

Text S1 Indirect method to ascertain reliability to dendrograms generated using ClaP method.
(DOCX)
