# Supplementary material for: Hybrid and Rogue Kinases Encoded in the Genomes of Model Eukaryotes
Source: PLoS One. 2014 Sep 25;9(9):e107956. doi: 10.1371/journal.pone.0107956 (PMC4177888; doi:10.1371/journal.pone.0107956)
Supplement: Figure S1 — Maximum likelihood trees showing various subfamilies that contain hybrid/rogue kinases with the canonical cases in black and hybrids highlighted in red and rogues are highlighted in green. Scale bars indicate distances as number of amino acid substitutions per site. A) Eph, B) Focal adhesion kinase, C) Met, D) Ror, E) Fer, F) PKC, G) Src H) PDGFR I) MAST and J) NDR. (PDF) [file pone.0107956.s001.pdf]

Figure S1: Maximum likelihood trees showing various subfamilies that contain hybrid/rogue kinases with the canonical cases in black and hybrids highlighted in red and rogues are highlighted in green. Scale bars indicate distances as number of amino acid substitutions per site. A)Eph, B) Focal adhesion kinase, C) Met, D) Ror, E) Fer, F) PKC, G) Src H) PDGFR I) MAST and J) NDR.

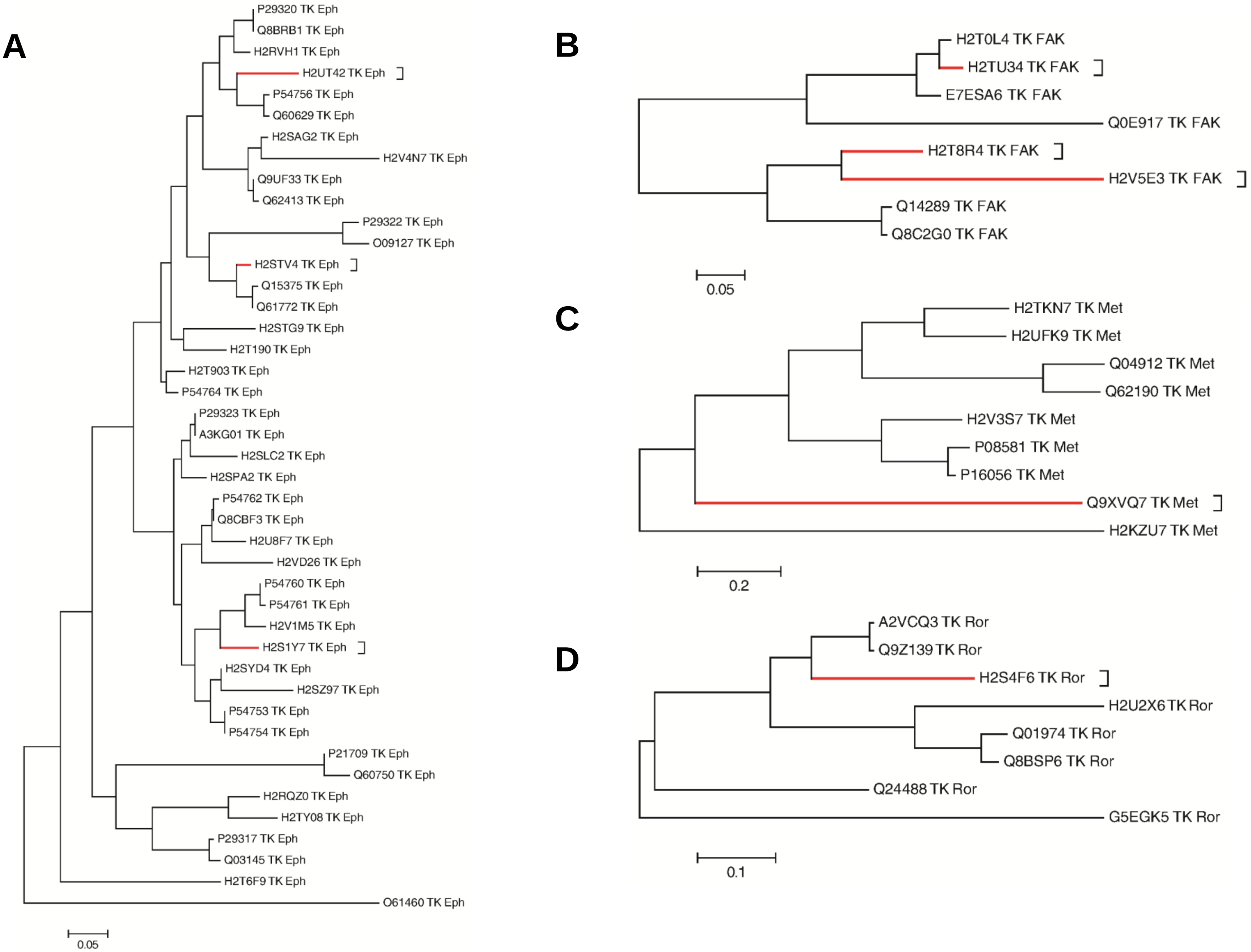

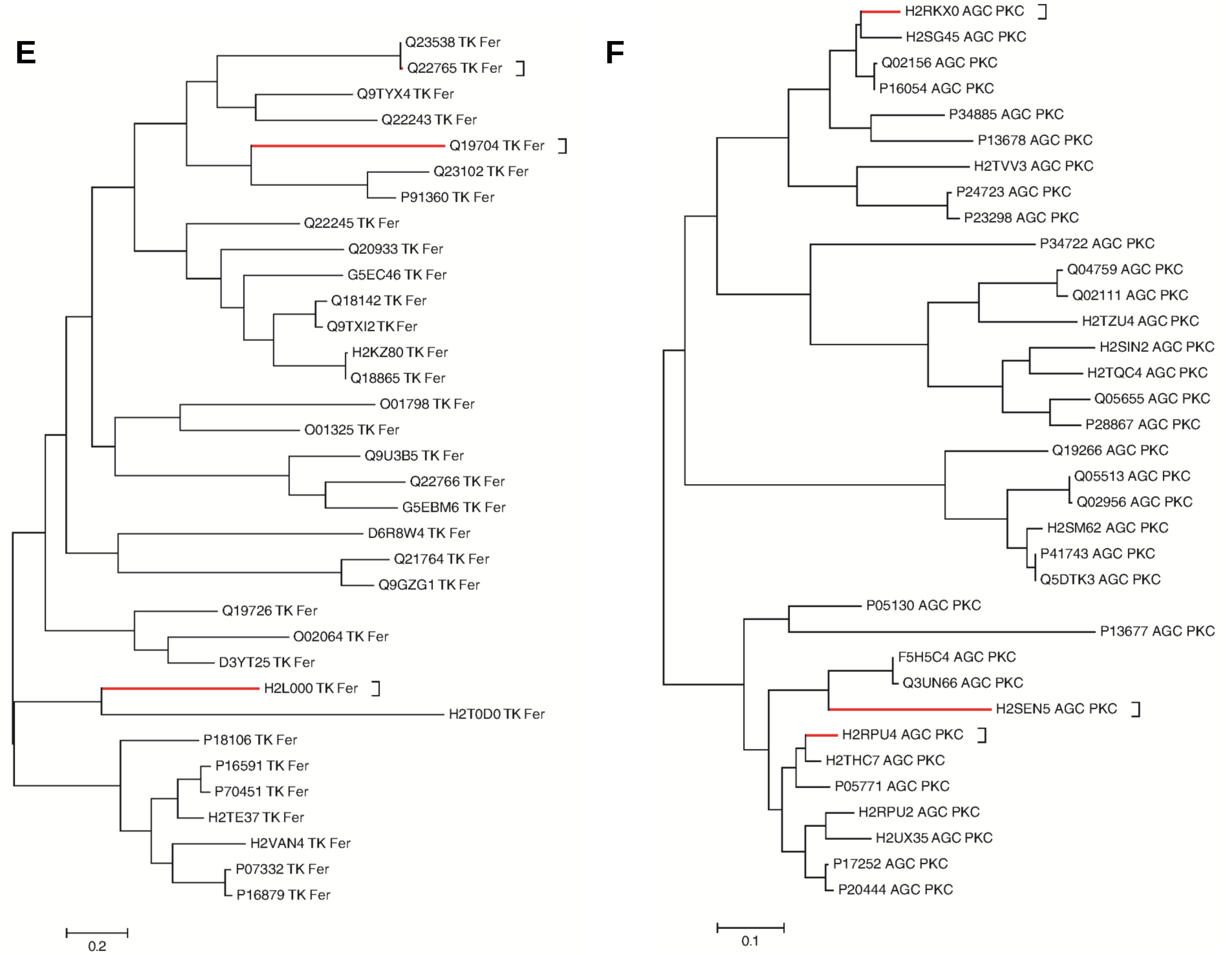

E
Q23538 TK Fer
Q22765 TK Fer
Q9TYX4 TK Fer
Q22243 TK Fer
Q19704 TK Fer
Q23102 TK Fer
P91360 TK Fer
Q22245 TK Fer
Q20933 TK Fer
G5EC46 TK Fer
Q18142 TK Fer
Q9TXI2 TK Fer
H2KZ80 TK Fer
Q18865 TK Fer
O01798 TK Fer
O01325 TK Fer
Q9U3B5 TK Fer
Q22766 TK Fer
G5EBM6 TK Fer
D6R8W4 TK Fer
Q21764 TK Fer
Q9GZG1 TK Fer
Q19726 TK Fer
O02064 TK Fer
D3YT25 TK Fer
H2L000 TK Fer
H2T0D0 TK Fer
P18106 TK Fer
P16591 TK Fer
P70451 TK Fer
H2TE37 TK Fer
H2VAN4 TK Fer
P07332 TK Fer
P16879 TK Fer
0.2
F
H2RKX0 AGC PKC
H2SG45 AGC PKC
Q02156 AGC PKC
P16054 AGC PKC
P34885 AGC PKC
P13678 AGC PKC
H2TVV3 AGC PKC
P24723 AGC PKC
P23298 AGC PKC
P34722 AGC PKC
Q04759 AGC PKC
Q02111 AGC PKC
H2TZU4 AGC PKC
H2SIN2 AGC PKC
H2TQC4 AGC PKC
Q05655 AGC PKC
P28867 AGC PKC
Q19266 AGC PKC
Q05513 AGC PKC
Q02956 AGC PKC
H2SM62 AGC PKC
P41743 AGC PKC
Q5DTK3 AGC PKC
P05130 AGC PKC
P13677 AGC PKC
F5H5C4 AGC PKC
Q3UN66 AGC PKC
H2SEN5 AGC PKC
H2RPU4 AGC PKC
H2THC7 AGC PKC
P05771 AGC PKC
H2RPU2 AGC PKC
H2UX35 AGC PKC
P17252 AGC PKC
P20444 AGC PKC
0.1

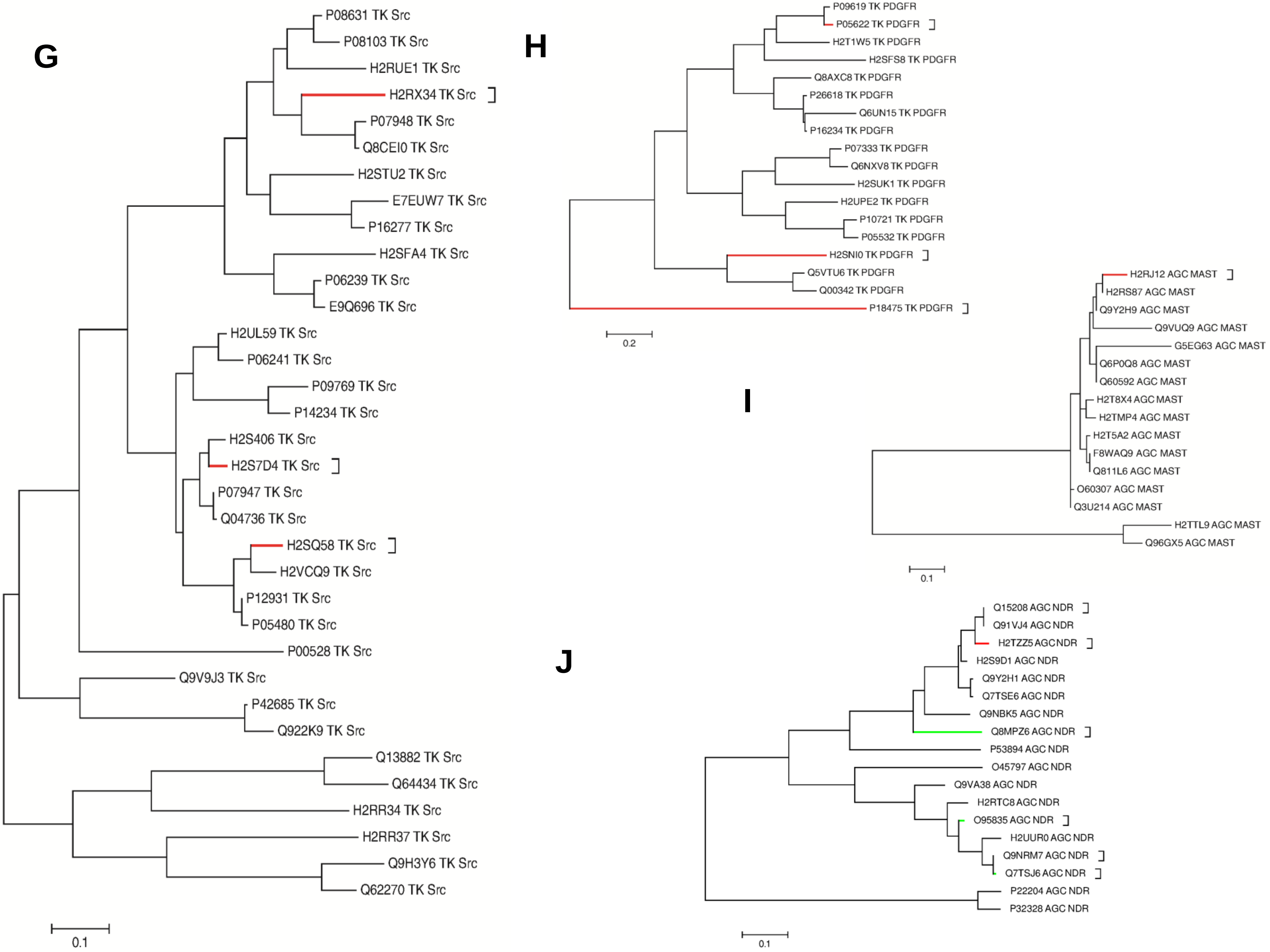
G
P08631 TK Src
P08103 TK Src
H2RUE1 TK Src
H2RX34 TK Src
P07948 TK Src
Q8CEI0 TK Src
H2STU2 TK Src
E7EUW7 TK Src
P16277 TK Src
H2SFA4 TK Src
P06239 TK Src
E9Q696 TK Src
H2UL59 TK Src
P06241 TK Src
P09769 TK Src
P14234 TK Src
H2S406 TK Src
H2S7D4 TK Src
P07947 TK Src
Q04736 TK Src
H2SQ58 TK Src
H2VCQ9 TK Src
P12931 TK Src
P05480 TK Src
P00528 TK Src
Q9V9J3 TK Src
P42685 TK Src
Q922K9 TK Src
Q13882 TK Src
Q64434 TK Src
H2RR34 TK Src
H2RR37 TK Src
Q9H3Y6 TK Src
Q62270 TK Src
0.1
H
P09619 TK PDGFR
P05622 TK PDGFR
H2T1W5 TK PDGFR
H2SFS8 TK PDGFR
Q8AXC8 TK PDGFR
P26618 TK PDGFR
Q6UN15 TK PDGFR
P16234 TK PDGFR
P07333 TK PDGFR
Q6NXV8 TK PDGFR
H2SUK1 TK PDGFR
H2UPE2 TK PDGFR
P10721 TK PDGFR
P05532 TK PDGFR
H2SNI0 TK PDGFR
Q5VTU6 TK PDGFR
Q00342 TK PDGFR
P18475 TK PDGFR
0.2
I
H2RJ12 AGC MAST
H2RS87 AGC MAST
Q9Y2H9 AGC MAST
Q9VUQ9 AGC MAST
G5EG63 AGC MAST
Q6P0Q8 AGC MAST
Q60592 AGC MAST
H2T8X4 AGC MAST
H2TMP4 AGC MAST
H2T5A2 AGC MAST
F8WAQ9 AGC MAST
Q811L6 AGC MAST
O60307 AGC MAST
Q3U214 AGC MAST
H2TTL9 AGC MAST
Q96GX5 AGC MAST
0.1
J
Q15208 AGC NDR
Q91VJ4 AGC NDR
H2TZZ5 AGC NDR
H2S9D1 AGC NDR
Q9Y2H1 AGC NDR
Q7TSE6 AGC NDR
Q9NBK5 AGC NDR
Q8MPZ6 AGC NDR
P53894 AGC NDR
O45797 AGC NDR
Q9VA38 AGC NDR
H2RTC8 AGC NDR
O95835 AGC NDR
H2UUR0 AGC NDR
Q9NRM7 AGC NDR
Q7TSJ6 AGC NDR
P22204 AGC NDR
P32328 AGC NDR
0.1
