# Supplementary material for: Hybrid and Rogue Kinases Encoded in the Genomes of Model Eukaryotes
Source: PLoS One. 2014 Sep 25;9(9):e107956. doi: 10.1371/journal.pone.0107956 (PMC4177888; doi:10.1371/journal.pone.0107956)
Supplement: Table S1 — Canonical domain architectures for each of the 91 subfamilies used in the study based on literature survey. (DOCX) [file pone.0107956.s002.docx]

Table S1. Canonical domain architectures for each of the 91 subfamilies used in the study based on literature survey.

| Subfamily | Domain combinations | | | References |
| --- | --- | --- | --- | --- |
| Abl | Kinase catalytic domain, SH3, SH2 and Factin_bind domains | | | [[1](#_ENREF_1)] |
| Ack | EBD,SAM,UBA | | | [[2](#_ENREF_2)] |
| AKT | PH, Kinase catalytic domain | | | [[3](#_ENREF_3)] |
| ALK | LDL_RECEPTOR, MAM | | | [[4](#_ENREF_4)] |
| Axl | Kinase catalytic domain, Ig,Fn3 | | | [[5](#_ENREF_5)] |
| CAMK1 | Single kinase domain | | | [[6](#_ENREF_6)] |
| CAMK2 | Single domain kinase | | | [[7](#_ENREF_7),[8](#_ENREF_8)] |
| CAMKL | | | In CAMKL, Kinase catalytic domain domain tethered to UBA [[8](#_ENREF_8)]  domain and long C-ter overhangs, KA1 | |
| CASK | Kinase catalytic domain, Calmodulin binding domain, PDZ/DHR,SH3,Guanylate kinase | | | [[9](#_ENREF_9)] |
| CCK4 | Kinase catalytic domain, Ig like domain | | | [[10](#_ENREF_10)] |
| CDK | Single domain kinases | | | [[11](#_ENREF_11),[12](#_ENREF_12)] |
| CDKL | Single domain kinases | | | [[13](#_ENREF_13)] |
| CK1 | Single domain kinases | | | [[14](#_ENREF_14)] |
| CK2 | Single domain kinases | | | [[13](#_ENREF_13)] |
| CLK | Single domain kinases | | | [[13](#_ENREF_13)] |
| Csk | Kinase catalytic domain,Sh3,SH2 | | | [[15](#_ENREF_15)] |
| DAPK | Single kinase domain. Rarely tethered to death domain and Ank repeats | | | [[16](#_ENREF_16)]  [[17](#_ENREF_17)] |
| DCAMKL | Either single kinase domain or associated with DCX domain | | | [[18](#_ENREF_18)] |
| DDR | Kinase catalytic domain, Ig like domains, F5-F8_type C domain | | | [[19](#_ENREF_19)] |
| DMPK | Kinase catalytic domain, coiled coil | | | [[20](#_ENREF_20)] |
| Dual | Has two tandem kinase domains | | | [[21](#_ENREF_21)] |
| DYRK | Single domain kinase | | | [[22](#_ENREF_22),[23](#_ENREF_23)] |
| EGFR | Recep_L, furin, Kinase catalytic domain | | | [[24](#_ENREF_24)] |
| Eph | Kinase catalytic domain Ephrin_lbd,, fn3, SAM, GCC2_GCC3,  Interfer-bind | | | [[25](#_ENREF_25)] |
| FAK | Kinase catalytic domain, FERN, Focal_adhesion domain | | | [[26](#_ENREF_26)] |
| Fer | Protein kinase and SH2 domain | | | [[27](#_ENREF_27)] |
| FGFR | Associated with different Ig family domains like Ig, Vset, Iset, | | | [[28](#_ENREF_28)] |
| GRK | Two domain kinase having Pk and RGS domain and soetimes PH | | | [[29](#_ENREF_29)] |
| GSK | Single domain kinases | | | [[30](#_ENREF_30)] |
| InsR | Kinase catalytic domain, fn3, furin-like and Recep-L-domain | | | [[31](#_ENREF_31)] |
| IRAK | Pkinse, death | | | [[32](#_ENREF_32)] |
| JakA | Has two tandem kinase domains | | | [[33](#_ENREF_33)] |
| KIN16 | Single Kinase catalytic domain domain | | | [[21](#_ENREF_21)] |
| KIN6 | Single Kinase catalytic domain omain | | | [[21](#_ENREF_21)] |
| LISK | | Mostly single Kinase catalytic domain domain, also associated with LIM [[34](#_ENREF_34)]  and PDZ domains [[35](#_ENREF_35)] | | |
| Lmr | Kinase catalytic domain and long Cterminal tail | | | [[36](#_ENREF_36)] |
| LRRK | LRR repeats, Ank repeats, Gtpase, COR,Kinase catalytic domain | | | [[37](#_ENREF_37)] |
| MAPK | Single domain kinases | | | [[30](#_ENREF_30)] |
| MAPKAPK | Single kinase domain | | | [[38](#_ENREF_38)] |
| MAST | Kinase catalytic domain,PDZ,DUF1908 | | | [[39](#_ENREF_39)] |
| Met | Kinase catalytic domain and PDZ | | | [[40](#_ENREF_40)] |
| MLCK | Kinase catalytic domain, Ig and fn3 | | | [[41](#_ENREF_41)] |
| MLK | Kinase catalytic domain, Ank repeats and SH3 | | | [[42](#_ENREF_42)] |
| Musk | Fz, Ig,Kinase catalytic domain | | | [[43](#_ENREF_43)] |
| NDR | Single kinase domain | | | [[44](#_ENREF_44)] |
| PDGFR | Kinase catalytic domain, Ig like domains | | | [[45](#_ENREF_45)] |
| PDK1 | Kinase catalytic domain and PH | | | [[46](#_ENREF_46)] |
| PHK | Single domain kinases | | | [[47](#_ENREF_47)] |
| PIM | Single domain kinases | | | [[48](#_ENREF_48)] |
| PKA | Single domain kinases | | | [[49](#_ENREF_49)] |
| PKC | Multi-domain kinase; Has C1,C2 and Pk domain,PB1,C1 and Kinase catalytic domain | | | [[50](#_ENREF_50)] |
| PKD | Kinase catalytic domain, C1, PH | | | [[51](#_ENREF_51)] |
| PKG | Kinase domain and cNMP binding domain | | | [[49](#_ENREF_49)] |
| PKN | HR1 repeats, Kinase catalytic domain_cterm, Kinase catalytic domain | | | [[52](#_ENREF_52)] |
| PSK | Kinase catalytic domain | | | [[53](#_ENREF_53)] |
| RAD53 | Kinase catalytic domain, FHA | | | [[54](#_ENREF_54)] |
| RAF | Kinase catalytic domain, CBD and RBD | | | [[55](#_ENREF_55)] |
| RCK | Single Kinase catalytic domain domain | | | [[56](#_ENREF_56)] |
| Ret | Cadherin,Kinase catalytic domain | | | [[57](#_ENREF_57)] |
| RIPK | Kinase catalytic domain,RHIM, death | | | [[58](#_ENREF_58)] |
| Ror | Ig,Fz,Kringle,Kinase catalytic domain | | | [[59](#_ENREF_59)] |
| RSK | Has two tandem kinase domains | | | [[60](#_ENREF_60)] |
| RSKL | Kinase catalytic domain,PX,MIT | | | [[49](#_ENREF_49)] |
| RSKR | single kinase domain | | | [[49](#_ENREF_49)] |
| Ryk | Kinase catalytic domain,WIF | | | [[61](#_ENREF_61)] |
| Sev | fn3,Kinase catalytic domain | | | [[62](#_ENREF_62)] |
| SGK | Mostly Single domain kinase; few have PX domain at the C-terminus | | | [[49](#_ENREF_49)] |
| Src | Kinase catalytic domain,Sh3,SH2 | | | [[15](#_ENREF_15)] |
| SRPK | Single domain kinases | | | [[13](#_ENREF_13)] |
| STE11 | Single kinase domain. Also associated with PB1 | | | [[63](#_ENREF_63)] |
| STE20 | Diverse domain combinations | | | [[64](#_ENREF_64)] |
| STE7 | Mainly single kinase domain, sometimes associated with PB1 | | | [[65](#_ENREF_65)] |
| STKR | Protein kinase, Activin_recp, TGF_beta_GS | | | [[66](#_ENREF_66)] |
| Syk | Kinase catalytic domain,SH2 | | | [[15](#_ENREF_15)] |
| Tec | Kinase catalytic domain, PH, Sh3, SH2 and BTK. | | | [[67](#_ENREF_67)] |
| Tie | Ig,EGFR modules,fn3,Kinase catalytic domain | | | [[68](#_ENREF_68)] |
| Trbl | Single kinase domain | | | [[69](#_ENREF_69)] |
| Trio | RhoGEF,PH,spectrin,Ig,Kinase catalytic domain | | | [[70](#_ENREF_70)] |
| Trk | Kinase catalytic domain, LRR, Lrrct1, Ig | | | [[71](#_ENREF_71)] |
| TSSK | single kinase domain | | | [[72](#_ENREF_72)] |
| TTBK | single kinase domain, filament | | | [[73](#_ENREF_73)] |
| TTBKL | Single kinase domain | | | [[73](#_ENREF_73)] |
| VEGFR | Kinase catalytic domain, Ig like domains | | | [[45](#_ENREF_45)] |
| VRK | Single kinase domain | | | [[74](#_ENREF_74)] |
| Worm10 | Single kinase domain | | | [[21](#_ENREF_21)] |
| Worm6 | Single kinase domain | | | [[21](#_ENREF_21)] |
| Worm7 | Single kinase domain | | | [[21](#_ENREF_21)] |
| Worm8 | Single kinase domain | | | [[21](#_ENREF_21)] |
| Worm9 | Single kinase domain | | | [[21](#_ENREF_21)] |
| YANK | Single domain kinases | | | [[75](#_ENREF_75)] |

1. Woodring PJ, Hunter T, Wang JY (2003) Regulation of F-actin-dependent processes by the Abl family of tyrosine kinases. Journal of cell science 116: 2613-2626.

2. Prieto-Echague V, Gucwa A, Brown DA, Miller WT (2010) Regulation of Ack1 localization and activity by the amino-terminal SAM domain. BMC biochemistry 11: 42.

3. Kohn AD, Takeuchi F, Roth RA (1996) Akt, a pleckstrin homology domain containing kinase, is activated primarily by phosphorylation. The Journal of biological chemistry 271: 21920-21926.

4. Palmer RH, Vernersson E, Grabbe C, Hallberg B (2009) Anaplastic lymphoma kinase: signalling in development and disease. The Biochemical journal 420: 345-361.

5. O'Bryan JP, Frye RA, Cogswell PC, Neubauer A, Kitch B, et al. (1991) axl, a transforming gene isolated from primary human myeloid leukemia cells, encodes a novel receptor tyrosine kinase. Molecular and cellular biology 11: 5016-5031.

6. Haribabu B, Hook SS, Selbert MA, Goldstein EG, Tomhave ED, et al. (1995) Human calcium-calmodulin dependent protein kinase I: cDNA cloning, domain structure and activation by phosphorylation at threonine-177 by calcium-calmodulin dependent protein kinase I kinase. The EMBO journal 14: 3679-3686.

7. Yang E, Schulman H (1999) Structural examination of autoregulation of multifunctional calcium/calmodulin-dependent protein kinase II. The Journal of biological chemistry 274: 26199-26208.

8. Kanaseki T, Ikeuchi Y, Sugiura H, Yamauchi T (1991) Structural features of Ca2+/calmodulin-dependent protein kinase II revealed by electron microscopy. The Journal of cell biology 115: 1049-1060.

9. Dimitratos SD, Woods DF, Bryant PJ (1997) Camguk, Lin-2, and CASK: novel membrane-associated guanylate kinase homologs that also contain CaM kinase domains. Mechanisms of development 63: 127-130.

10. Grassot J, Gouy M, Perriere G, Mouchiroud G (2006) Origin and molecular evolution of receptor tyrosine kinases with immunoglobulin-like domains. Molecular biology and evolution 23: 1232-1241.

11. Russo AA, Jeffrey PD, Pavletich NP (1996) Structural basis of cyclin-dependent kinase activation by phosphorylation. Nature structural biology 3: 696-700.

12. Brown NR, Noble ME, Endicott JA, Johnson LN (1999) The structural basis for specificity of substrate and recruitment peptides for cyclin-dependent kinases. Nature cell biology 1: 438-443.

13. Kannan N, Neuwald AF (2004) Evolutionary constraints associated with functional specificity of the CMGC protein kinases MAPK, CDK, GSK, SRPK, DYRK, and CK2alpha. Protein science : a publication of the Protein Society 13: 2059-2077.

14. Hanks SK, Hunter T (1995) Protein kinases 6. The eukaryotic protein kinase superfamily: kinase (catalytic) domain structure and classification. FASEB journal : official publication of the Federation of American Societies for Experimental Biology 9: 576-596.

15. Parsons SJ, Parsons JT (2004) Src family kinases, key regulators of signal transduction. Oncogene 23: 7906-7909.

16. Shohat G, Spivak-Kroizman T, Cohen O, Bialik S, Shani G, et al. (2001) The pro-apoptotic function of death-associated protein kinase is controlled by a unique inhibitory autophosphorylation-based mechanism. The Journal of biological chemistry 276: 47460-47467.

17. Tian JH, Das S, Sheng ZH (2003) Ca2+-dependent phosphorylation of syntaxin-1A by the death-associated protein (DAP) kinase regulates its interaction with Munc18. The Journal of biological chemistry 278: 26265-26274.

18. Horesh D, Sapir T, Francis F, Wolf SG, Caspi M, et al. (1999) Doublecortin, a stabilizer of microtubules. Human molecular genetics 8: 1599-1610.

19. Exposito JY, Larroux C, Cluzel C, Valcourt U, Lethias C, et al. (2008) Demosponge and sea anemone fibrillar collagen diversity reveals the early emergence of A/C clades and the maintenance of the modular structure of type V/XI collagens from sponge to human. The Journal of biological chemistry 283: 28226-28235.

20. Garcia P, Ucurum Z, Bucher R, Svergun DI, Huber T, et al. (2006) Molecular insights into the self-assembly mechanism of dystrophia myotonica kinase. FASEB journal : official publication of the Federation of American Societies for Experimental Biology 20: 1142-1151.

21. Manning G (2005) Genomic overview of protein kinases. WormBook : the online review of C elegans biology: 1-19.

22. Becker W, Weber Y, Wetzel K, Eirmbter K, Tejedor FJ, et al. (1998) Sequence characteristics, subcellular localization, and substrate specificity of DYRK-related kinases, a novel family of dual specificity protein kinases. The Journal of biological chemistry 273: 25893-25902.

23. Becker W, Joost HG (1999) Structural and functional characteristics of Dyrk, a novel subfamily of protein kinases with dual specificity. Progress in nucleic acid research and molecular biology 62: 1-17.

24. Arkhipov A, Shan Y, Das R, Endres NF, Eastwood MP, et al. (2013) Architecture and membrane interactions of the EGF receptor. Cell 152: 557-569.

25. Kullander K, Klein R (2002) Mechanisms and functions of Eph and ephrin signalling. Nature reviews Molecular cell biology 3: 475-486.

26. Karginov AV, Ding F, Kota P, Dokholyan NV, Hahn KM (2010) Engineered allosteric activation of kinases in living cells. Nature biotechnology 28: 743-747.

27. Kim L, Wong TW (1998) Growth factor-dependent phosphorylation of the actin-binding protein cortactin is mediated by the cytoplasmic tyrosine kinase FER. The Journal of biological chemistry 273: 23542-23548.

28. Olsen SK, Ibrahimi OA, Raucci A, Zhang F, Eliseenkova AV, et al. (2004) Insights into the molecular basis for fibroblast growth factor receptor autoinhibition and ligand-binding promiscuity. Proceedings of the National Academy of Sciences of the United States of America 101: 935-940.

29. Singh P, Wang B, Maeda T, Palczewski K, Tesmer JJ (2008) Structures of rhodopsin kinase in different ligand states reveal key elements involved in G protein-coupled receptor kinase activation. The Journal of biological chemistry 283: 14053-14062.

30. Tanoue T, Nishida E (2003) Molecular recognitions in the MAP kinase cascades. Cellular signalling 15: 455-462.

31. Menting JG, Ward CW, Margetts MB, Lawrence MC (2009) A thermodynamic study of ligand binding to the first three domains of the human insulin receptor: relationship between the receptor alpha-chain C-terminal peptide and the site 1 insulin mimetic peptides. Biochemistry 48: 5492-5500.

32. Wang Z, Liu J, Sudom A, Ayres M, Li S, et al. (2006) Crystal structures of IRAK-4 kinase in complex with inhibitors: a serine/threonine kinase with tyrosine as a gatekeeper. Structure 14: 1835-1844.

33. Kisseleva T, Bhattacharya S, Braunstein J, Schindler CW (2002) Signaling through the JAK/STAT pathway, recent advances and future challenges. Gene 285: 1-24.

34. Ranganathan R, Ross EM (1997) PDZ domain proteins: scaffolds for signaling complexes. Current biology : CB 7: R770-773.

35. Bach I (2000) The LIM domain: regulation by association. Mechanisms of development 91: 5-17.

36. Wang H, Brautigan DL (2002) A novel transmembrane Ser/Thr kinase complexes with protein phosphatase-1 and inhibitor-2. The Journal of biological chemistry 277: 49605-49612.

37. Drolet RE, Sanders JM, Kern JT (2011) Leucine-rich repeat kinase 2 (LRRK2) cellular biology: a review of recent advances in identifying physiological substrates and cellular functions. Journal of neurogenetics 25: 140-151.

38. Meng W, Swenson LL, Fitzgibbon MJ, Hayakawa K, Ter Haar E, et al. (2002) Structure of mitogen-activated protein kinase-activated protein (MAPKAP) kinase 2 suggests a bifunctional switch that couples kinase activation with nuclear export. The Journal of biological chemistry 277: 37401-37405.

39. Lumeng C, Phelps S, Crawford GE, Walden PD, Barald K, et al. (1999) Interactions between beta 2-syntrophin and a family of microtubule-associated serine/threonine kinases. Nature neuroscience 2: 611-617.

40. Gherardi E, Youles ME, Miguel RN, Blundell TL, Iamele L, et al. (2003) Functional map and domain structure of MET, the product of the c-met protooncogene and receptor for hepatocyte growth factor/scatter factor. Proceedings of the National Academy of Sciences of the United States of America 100: 12039-12044.

41. Kudryashov DS, Stepanova OV, Vilitkevich EL, Nikonenko TA, Nadezhdina ES, et al. (2004) Myosin light chain kinase (210 kDa) is a potential cytoskeleton integrator through its unique N-terminal domain. Experimental cell research 298: 407-417.

42. Durkin JT, Holskin BP, Kopec KK, Reed MS, Spais CM, et al. (2004) Phosphoregulation of mixed-lineage kinase 1 activity by multiple phosphorylation in the activation loop. Biochemistry 43: 16348-16355.

43. Till JH, Becerra M, Watty A, Lu Y, Ma Y, et al. (2002) Crystal structure of the MuSK tyrosine kinase: insights into receptor autoregulation. Structure 10: 1187-1196.

44. Takamori M (2012) Structure of the neuromuscular junction: function and cooperative mechanisms in the synapse. Annals of the New York Academy of Sciences 1274: 14-23.

45. Kazlauskas A, Cooper JA (1989) Autophosphorylation of the PDGF receptor in the kinase insert region regulates interactions with cell proteins. Cell 58: 1121-1133.

46. Dittrich AC, Devarenne TP (2012) Perspectives in PDK1 evolution: insights from photosynthetic and non-photosynthetic organisms. Plant signaling & behavior 7: 642-649.

47. Brushia RJ, Walsh DA (1999) Phosphorylase kinase: the complexity of its regulation is reflected in the complexity of its structure. Frontiers in bioscience : a journal and virtual library 4: D618-641.

48. Qian KC, Wang L, Hickey ER, Studts J, Barringer K, et al. (2005) Structural basis of constitutive activity and a unique nucleotide binding mode of human Pim-1 kinase. The Journal of biological chemistry 280: 6130-6137.

49. Pearce LR, Komander D, Alessi DR (2010) The nuts and bolts of AGC protein kinases. Nature reviews Molecular cell biology 11: 9-22.

50. Balendran A, Biondi RM, Cheung PC, Casamayor A, Deak M, et al. (2000) A 3-phosphoinositide-dependent protein kinase-1 (PDK1) docking site is required for the phosphorylation of protein kinase Czeta (PKCzeta ) and PKC-related kinase 2 by PDK1. The Journal of biological chemistry 275: 20806-20813.

51. Zhang X, Zhang S, Yamane H, Wahl R, Ali A, et al. (2006) Kinetic mechanism of AKT/PKB enzyme family. The Journal of biological chemistry 281: 13949-13956.

52. Shibata H, Mukai H, Inagaki Y, Homma Y, Kimura K, et al. (1996) Characterization of the interaction between RhoA and the amino-terminal region of PKN. FEBS letters 385: 221-224.

53. Schlafli P, Borter E, Spielmann P, Wenger RH (2009) The PAS-domain kinase PASKIN: a new sensor in energy homeostasis. Cellular and molecular life sciences : CMLS 66: 876-883.

54. Schwartz MF, Lee SJ, Duong JK, Eminaga S, Stern DF (2003) FHA domain-mediated DNA checkpoint regulation of Rad53. Cell cycle 2: 384-396.

55. Rushworth LK, Hindley AD, O'Neill E, Kolch W (2006) Regulation and role of Raf-1/B-Raf heterodimerization. Molecular and cellular biology 26: 2262-2272.

56. Dahlkvist A, Sunnerhagen P (1994) Two novel deduced serine/threonine protein kinases from Saccharomyces cerevisiae. Gene 139: 27-33.

57. Anders J, Kjar S, Ibanez CF (2001) Molecular modeling of the extracellular domain of the RET receptor tyrosine kinase reveals multiple cadherin-like domains and a calcium-binding site. The Journal of biological chemistry 276: 35808-35817.

58. Weinlich R, Dillon CP, Green DR (2011) Ripped to death. Trends in cell biology 21: 630-637.

59. Roszmusz E, Patthy A, Trexler M, Patthy L (2001) Localization of disulfide bonds in the frizzled module of Ror1 receptor tyrosine kinase. The Journal of biological chemistry 276: 18485-18490.

60. Dummler BA, Hauge C, Silber J, Yntema HG, Kruse LS, et al. (2005) Functional characterization of human RSK4, a new 90-kDa ribosomal S6 kinase, reveals constitutive activation in most cell types. The Journal of biological chemistry 280: 13304-13314.

61. Patthy L (2000) The WIF module. Trends in biochemical sciences 25: 12-13.

62. Mullins MC, Rubin GM (1991) Isolation of temperature-sensitive mutations of the tyrosine kinase receptor sevenless (sev) in Drosophila and their use in determining its time of action. Proceedings of the National Academy of Sciences of the United States of America 88: 9387-9391.

63. Nakamura K, Johnson GL (2007) Noncanonical function of MEKK2 and MEK5 PB1 domains for coordinated extracellular signal-regulated kinase 5 and c-Jun N-terminal kinase signaling. Molecular and cellular biology 27: 4566-4577.

64. Drogen F, O'Rourke SM, Stucke VM, Jaquenoud M, Neiman AM, et al. (2000) Phosphorylation of the MEKK Ste11p by the PAK-like kinase Ste20p is required for MAP kinase signaling in vivo. Current biology : CB 10: 630-639.

65. Gartner A, Nasmyth K, Ammerer G (1992) Signal transduction in Saccharomyces cerevisiae requires tyrosine and threonine phosphorylation of FUS3 and KSS1. Genes & development 6: 1280-1292.

66. Massague J, Weis-Garcia F (1996) Serine/threonine kinase receptors: mediators of transforming growth factor beta family signals. Cancer surveys 27: 41-64.

67. Wang DS, Shaw G (1995) The association of the C-terminal region of beta I sigma II spectrin to brain membranes is mediated by a PH domain, does not require membrane proteins, and coincides with a inositol-1,4,5 triphosphate binding site. Biochemical and biophysical research communications 217: 608-615.

68. Macdonald PR, Progias P, Ciani B, Patel S, Mayer U, et al. (2006) Structure of the extracellular domain of Tie receptor tyrosine kinases and localization of the angiopoietin-binding epitope. The Journal of biological chemistry 281: 28408-28414.

69. Hegedus Z, Czibula A, Kiss-Toth E (2007) Tribbles: a family of kinase-like proteins with potent signalling regulatory function. Cellular signalling 19: 238-250.

70. Debant A, Serra-Pages C, Seipel K, O'Brien S, Tang M, et al. (1996) The multidomain protein Trio binds the LAR transmembrane tyrosine phosphatase, contains a protein kinase domain, and has separate rac-specific and rho-specific guanine nucleotide exchange factor domains. Proceedings of the National Academy of Sciences of the United States of America 93: 5466-5471.

71. Ultsch MH, Wiesmann C, Simmons LC, Henrich J, Yang M, et al. (1999) Crystal structures of the neurotrophin-binding domain of TrkA, TrkB and TrkC. Journal of molecular biology 290: 149-159.

72. Makarova KS, Aravind L, Galperin MY, Grishin NV, Tatusov RL, et al. (1999) Comparative genomics of the Archaea (Euryarchaeota): evolution of conserved protein families, the stable core, and the variable shell. Genome research 9: 608-628.

73. Sato S, Cerny RL, Buescher JL, Ikezu T (2006) Tau-tubulin kinase 1 (TTBK1), a neuron-specific tau kinase candidate, is involved in tau phosphorylation and aggregation. Journal of neurochemistry 98: 1573-1584.

74. Fu T, Ren H, Zhang J, Ren P, Enyedy I, et al. (2013) Role of bivalent cations in structural stabilities of new drug targets--vaccinia-related kinases (VRK) from molecular dynamics simulations. Current pharmaceutical design 19: 2269-2281.

75. Arencibia JM, Pastor-Flores D, Bauer AF, Schulze JO, Biondi RM (2013) AGC protein kinases: from structural mechanism of regulation to allosteric drug development for the treatment of human diseases. Biochimica et biophysica acta 1834: 1302-1321.
