# Supplementary material for: Hybrid and Rogue Kinases Encoded in the Genomes of Model Eukaryotes
Source: PLoS One. 2014 Sep 25;9(9):e107956. doi: 10.1371/journal.pone.0107956 (PMC4177888; doi:10.1371/journal.pone.0107956)
Supplement: Table S2 — List of hybrid and rogue kinases from the six model eukaryotes S. cerevisiae, C.elegans, D.melanogaster, T.rubripes, M.musmusculus, H.sapiens. (DOCX) [file pone.0107956.s003.docx]

Table S2. List of hybrid and rogue kinases from the six model eukaryotes *S. cerevisiae, C.elegans, D.melanogaster, T.rubripes, M.musmusculus, H.sapiens*.

| Sequence | Unusuality | Orphans | Hybrid/rogue | Organism | Notes | | Reference |
| --- | --- | --- | --- | --- | --- | --- | --- |
| C0Z1Y5_CMGC_CLK | Extra SPK domain | * | Rogue | C.ele | Found only in C.elegans. Importance highlighted in metabolic, developmental, or evolutionary processes that are unique to Caenorhabditis species | | [[1](#_ENREF_1)] |
| Q19243_CMGC_MAPK | Extra His_Phos_1 domain | * | Rogue | C.ele | The His phosphatase is seen in fructokinase bi-functional enzyme where it enables catalyse | | [[2](#_ENREF_2)] |
| E9JGM7_CAMK_DAPK | Extra Filament domain |  | Hybrid | C.ele | Seen in primate lineage in Tau tubulin kinase and domain helps in localization to cytoskeleton/nuclear envelope region. This kinase has Zip kinase like properties and involved in membrane blebbing. | | [[3](#_ENREF_3)] |
| H2U7P7_CAMK_DAPK | Extra Filament domain |  | Hybrid | Fugu | May be involved in cilagenesis | | [[4](#_ENREF_4)], [[5](#_ENREF_5)] |
| E9QM23_CAMK_CASK | Extra L27 domain | * | Rogue | Mouse | L27 is an adapter protein involved in unique protein-protein interactions. Only in 2002 was this domain first reported in a CASK Kinase catalytic domain as a novel domain with its function in protein-protein interactions | | [[6](#_ENREF_6)] |
| O14936_CAMK_CASK | Extra SH3 domain |  | Hybrid | Human | Protein-protein interactions with polyproline regions | |  |
| H2SXX6_CAMK_MLCK | Single Kinase catalytic domain MLCK |  | Hybrid | Fugu | Ligand binding altered | |  |
| H2TWD0_CAMK_MLCK | Single Kinase catalytic domain MLCK |  | Hybrid | Fugu | Ligand binding altered | |  |
| Q23260_CAMK_MLCK | Single Kinase catalytic domain MLCK |  | Hybrid | C.ele | Ligand binding altered | |  |
| H2T1I5_CAMK_CAMK2 | Extra SnoaL_2 domain | * | Rogue | Fugu | SnoaL is a polyketide cyclase predicted to be part of MAPK pathways in certain fungal species. Thus, by being tethered to CAMKL kinase leads to cross-talk | | [[7](#_ENREF_7)], [[8](#_ENREF_8)] |
| P24719_CAMK_CAMK1 | Extra FHA domain | * | Hybrid | Yeast |  | |  |
| E9Q767_CAMK_CAMKL | Extra Reprolysin,Pep_M12B_propep and Disintegrin domain | * | Rogue | Mouse | Reprolysin is a peptidase and disintegrin is found in snake venom. A similar sequence is reported in water sponge associated with tyrosine kinase and is electronically annotated to be a proteolytic enzyme located at cell memebrane. | [[9](#_ENREF_9)] | |
| Q8CEE6_CAMK_CAMKL | Extra PAS domain |  | Rogue | Mouse | Sensory function in energy homeostasis and protein translation | | [[10](#_ENREF_10),[11](#_ENREF_11)] |
| Q96RG2_CAMK_CAMKL | Extra PAS domain |  | Rogue | Human | Sensory function in energy homeostasis and protein translation | | [[10](#_ENREF_10),[11](#_ENREF_11)] |
| O94806_CAMK_PKD | Extra Zf_ring like domain | * | Rogue | Human | Role in cytoskeleton remodelling | | [[12](#_ENREF_12)] |
| O75962_CAMK_Trio | Extra SH3 domain |  | Hybrid | Human | Protein-protein interactions with polyproline regions | |  |
| O60229_CAMK_Trio | Extra SH3 domain |  | Hybrid | Human | Protein-protein interactions with polyproline regions | |  |
| H2L000_TK_Fer | Single Kinase catalytic domain Fer |  | Hybrid | C.ele | The localization and substrate specificities of this kinase is likely to be affected. | | [[13](#_ENREF_13)] |
| H2T0D0_TK_Fer | Single kinase Fer |  | Hybrid | Fugu | The localization and substrate specificities of this kinase is likely to be affected. | | [[13](#_ENREF_13)] |
| Q22765_TK_Fer | Single Kinase catalytic domain Fer |  | Hybrid | Caeel | The localization and substrate specificities of this kinase is likely to be affected. | |  |
| Q19704_TK_Fer | Single Kinase catalytic domain Fer |  | Hybrid | Caeel | The localization and substrate specificities of this kinase is likely to be affected. | |  |
| H2RX34_TK_Src | Single Kinase catalytic domain Src |  | Hybrid | Fugu | The localization and substrate specificities of this kinase is likely to be affected. | |  |
| H2S7D4_TK_Src | Single Kinase catalytic domain Src |  | Hybrid | Fugu | The localization and substrate specificities of this kinase is likely to be affected. | |  |
| H2SQ58_TK_Src | Single Kinase catalytic domain Src |  | Hybrid | Fugu | The localization and substrate specificities of this kinase is likely to be affected. | |  |
| H2S1Y7_TK_Eph | Single Pkinse Eph |  | Hybrid | Fugu | Not likely to function as a receptor. Cytosolic tyrosine kinase | | [[14](#_ENREF_14)] |
| H2UT42_TK_Eph | Single Kinase catalytic domain Eph |  | Hybrid | Fugu | Not likely to function as a receptor. Cytosolic tyrosine kinase | |  |
| H2STV4_TK_Eph | Single Kinase catalytic domain Eph |  | Hybrid | Fugu | Not likely to function as a receptor. Cytosolic tyrosine kinase | |  |
| H2S4F6_TK_Ror | Single Kinase catalytic domain Ror | * | Hybrid | Fugu | Not likely to function as a receptor. Cytosolic tyrosine kinase | | [[15](#_ENREF_15)] |
| H2S653_TK_Musk | Extra Kringle domain | * | Hybrid | Fugu | Its a protein protein interaction domain. This specific combination resembles that of Ror kinase | | |
| H2TU34_TK_FAK | Single Kinase catalytic domain FAK |  | Hybrid | Fugu | Localization and binding specificities altered. | | [[16](#_ENREF_16)] |
| H2V5E3_TK_FAK | Single Kinase catalytic domain FAK |  | Hybrid | Fugu | Localization and binding specificities altered. | | [[16](#_ENREF_16)] |
| H2T8R4_TK_FAK | Single Kinase catalytic domain FAK |  | Hybrid | Fugu | Localization and binding specificities altered. | | [[16](#_ENREF_16)] |
| P03949_TK_Abl | No F-actn domain |  | Hybrid | Caeel | Factn bind helps retain in cytosol, therefore localization altered. | | [[17](#_ENREF_17)] |
| H2SNI0_TK_PDGFR | Single Kinase catalytic domain domain |  | Hybrid | Fugu | Not likely to function as a receptor. Cytosolic tyrosine kinase | |  |
| P05622_TK_PDGFR | Single Kinase catalytic domain domain |  | Hybrid | Mouse | Not likely to function as a receptor. Cytosolic tyrosine kinase | |  |
| P18475_TK_PDGFR | Single Kinase catalytic domain domain |  | Hybrid | Drome | Altered ligand binding properties | |  |
| P16234_TK_PDGFR | Herpes_gE domain instead of Ig domain |  | Hybrid | Human | Altered ligand binding properties | |  |
| P21802_TK_FGFR | Herpes_gE domain instead of I-set domain |  | Hybrid | Human | Altered ligand binding properties | |  |
| E9PFZ5_TK_CCK4 | Herpes_gE domain instead of I-set domain |  | Hybrid | Human | Altered ligand binding properties | |  |
| Q16832_TK_DDR | Herpes_gE domain instead of Ig domain |  | Hybrid | Human | Altered ligand binding properties | |  |
| P29376_TK_ALK | No Recep_L and MAM domain but Gly-rich domain extra | * | Hybrid | Human | The MAM domain in ALK helps it localise to membrane and serves a receptor molecule. Therefore, localization is likely to be affected. | | [[18](#_ENREF_18)], [[19](#_ENREF_19)], [[20](#_ENREF_20)] |
| Q0VGZ5_TK_ALK | Single Kinase catalytic domain ALK |  | Hybrid | Mouse | The MAM domain in ALK helps it localise to membrane and serves a receptor molecule. Therefore, localization is likely to be affected. | |  |
| O76695_TK_KIN16 | Extra ZZ domain | * | Rogue | Caeel | Single zz occurs in transcription adaptors. So, its likely to have role in transcription | [[21](#_ENREF_21)] | |
| Q21038_TK_KIN16 | Extra Ig like domains |  | Hybrid | Caeel | Altered ligand binding properties | |  |
| Q21041_TK_KIN16 | Extra Ig like domains |  | Hybrid | Caeel | Altered ligand binding properties | |  |
| Q24145_TK_Syk | Extra Ank repeats | * | Hybrid | Drome | Regulates signalling of epithelial cell polarity | | [[22](#_ENREF_22)] |
| Q9XVQ7_TK_Met | Single Kinase catalytic domain Met | * | Hybrid | Caeel | The PDZ domain helps in forming signallosome complexes. So, complexation is likely to be affected or maybe compensated by other binding proteins | | [[23](#_ENREF_23)] |
| H2SGC1_TKL_RAF | Extra Gal-bind-lectin | * | Rogue | Fugu | RAF activates MAPK pathway. Relate this to functions of gal bind lectin in wiki |  | |
| Q16671_TKL_STKR | Extra RELT domain | * | Rogue | Human | Associated with TNF related pathways. | | [[24](#_ENREF_24)] |
| H2TDZ8_TKL_MLK | Extra IFT57 | * | Hybrid | Fugu | Required for maintenance of cilia/flagella. Some cases found in association of NimA kinases in Chlamydomonas | | [[25](#_ENREF_25)] |
| H2V7M0_TKL_MLK | Extra SAM1 domain |  | Hybrid | Fugu | Important for dimerization | | [[26](#_ENREF_26)] |
| P53671_TKL_LISK | Extra PPI-inhibitor domain | * | Rogue | Human | Contraction of vascular smooth muscles | | [[27](#_ENREF_27)] |
| Q9ESL4_TKL_MLK | No Ank,SH3 but SAM1 domain |  | Hybrid | Mouse | Induces dimerization | | [[28](#_ENREF_28)] |
| Q5TCY1_CK1_TTBK | Extra DAXX domain | * | Rogue | Human | Role in transcriptional regulation. | | [[29](#_ENREF_29)] |
| H2TM27_STE_STE11 | Extra Myosin_TH1 | * | Hybrid | Fugu | Certain motifs in TH1 domain required for membrane localization | | [[30](#_ENREF_30)] |
| P23561_STE_STE11 | Extra SAM_2 domain |  | Hybrid | Yeast | Required for dimerization with STE50 | | [[31](#_ENREF_31)] |
| P53349_STE_STE11 | Extra zf-rbx1 domain |  | Rogue | Mouse | Participates in cell cycle and chromosome metabolism. Binding to DNA, RNA or metal ions. | | [[32](#_ENREF_32)],  [[33](#_ENREF_33)] |
| Q13233_STE_STE11 | Extra zf-rbx1 domain |  | Rogue | Human | Participates in cell cycle and chromosome metabolism.Binding to DNA, RNA or metal ions. | | [[32](#_ENREF_32),[33](#_ENREF_33)] |
| O75914_STE_STE20 | Extra Ribosomal_60s domain | * | Rogue | Human | probably regulated by RNA binding | |  |
| H2T8K6_AGC_RSK | Single kinase RSK |  | Hybrid | Fugu | Single kinase RSK. RSK has CTD kinase domain which activates the RSK. Therefore its likely to have a different activation mechanism | | [[34](#_ENREF_34)] |
| P53739_AGC_RSK | Single Kinase catalytic domain RSK |  | Hybrid | Yeast | Called flippase in yeast which is externally regulated by another kinase called Ypk1 | | [[35](#_ENREF_35)] |
| Q19858_AGC_RSK | Single Kinase catalytic domain RSK |  | Hybrid | Caeel | Single kinase RSK. RSK has CTD kinase domain which activates the RSK. Therefore its likely to have a different activation mechanism | | [[34](#_ENREF_34)] |
| Q9UBS0_AGC_RSK | Single Kinase catalytic domain RSK |  | Hybrid | Human | RSK has CTD kinase domain which activates the RSK. Therefore its likely to have a different activation mechanism | | [[34](#_ENREF_34)] |
| Q9Z1M4_AGC_RSK | Single Kinase catalytic domain RSK |  | Hybrid | Mouse | RSK has CTD kinase domain which activates the RSK. Therefore its likely to have a different activation mechanism | | [[34](#_ENREF_34)] |
| H2RNW3_AGC_RSK | Single kinase RSK |  | Hybrid | Fugu | RSK has CTD kinase domain which activates the RSK. Therefore its likely to have a different activation mechanism | | [[34](#_ENREF_34)] |
| H2RJ12_AGC_MAST | Single Kinase catalytic domain MAST | * | Hybrid | Fugu | Single kinase domain MAST although of much longer length are reported as rare but prevalent which act as phosphatase inhibiters and different from classical MAST which act associate with phosphatases via PDZ domain to catalyse dephosphorylation reactions | | 20818157 |
| H2RKX0_AGC_PKC | Single Kinase catalytic domain PKC |  | Hybrid | Fugu | Single kinase PKC, and hence not likely to interact with DAG and Calcium | | [[34](#_ENREF_34)] |
| H2RPU4_AGC_PKC | Single Kinase catalytic domain PKC |  | Hybrid | Fugu | Single kinase PKC, and hence not likely to interact with DAG and Calcium | | [[34](#_ENREF_34)] |
| H2SEN5_AGC_PKC | Single Kinase catalytic domain PKC |  | Hybrid | Fugu | Single kinase PKC, and hence not likely to interact with DAG and Calcium | | [[34](#_ENREF_34)] |
| H2TZZ5_AGC_NDR | Single Kinase catalytic domain NDR | * | Hybrid | Fugu | Regulation likely to be different | |  |
| O95835_AGC_NDR | Extra UBA domain |  | Hybrid | Human | Connection to ubiquitination pathway | | [[36](#_ENREF_36)] |
| Q7TSJ6_AGC_NDR | Extra UBA domain |  | Hybrid | Mouse | Connection to ubiquitination pathway | |  |
| Q8MPZ6_AGC_NDR | Extra C1 and C2 domain | * | Hybrid | Caeel | Likely to be regulated by Ca^+2^, DAG | | [[37](#_ENREF_37)] |
| Q9NRM7_AGC_NDR | Extra UBA domain |  | Hybrid | Human | Cross-talk with Ubiquitination pathway. | |  |
| Q15208_AGC_NDR | Extra Alpha_MRAP domain | * | Rogue | Human | The alpha-2-macroglobulin receptor-associated protein (RAP) is a intracellular glycoprotein that binds to the 2-macroglobulin receptor and other members of the low density lipoprotein receptor family. | | [[38](#_ENREF_38)] |
| P11792_AGC_AKT | No PH domain |  | Hybrid | Yeast | Likely to localised in cytosol | |  |
| P12688_AGC_AKT | No PH domain |  | Hybrid | Yeast | Likely to localised in cytosol | |  |
| P18961_AGC_AKT | No PH domain |  | Hybrid | Yeast | Likely to localised in cytosol | |  |
| Q5VT25_AGC_DMPK | No PH domain |  | Hybrid | Human | Likely to localised in cytosol | |  |
| P49025_AGC_DMPK | No PBD |  | Hybrid | Mouse | Binding to Rho affected | |  |
| O75116_AGC_DMPK | Extra Rho-bind domain without PBD,C1 and CNH |  | Hybrid | Human | This domain architecture compensates for the absence of CNH and PBD domains. However, binding specificities and regulation are likely to be altered. | | [[39](#_ENREF_39),[40](#_ENREF_40)] |
| Q9VXE3_AGC_DMPK | Extra Rho-bind domain instead of CNH,DMPK-coil, PH and PBD |  | Hybrid | Drome | This domain architecture compensates for the absence of CNH and PBD domains. However, binding specificities are likely to be altered. | | [[39](#_ENREF_39),[40](#_ENREF_40)] |
| P70335_AGC_DMPK | Extra Rho-bind,HR1,Myosin-tail domain and no CNH and PBD |  | Hybrid | Mouse | This domain architecture compensates for the absence of CNH and PBD domains. However, binding specificities are likely to be altered. | | [[39](#_ENREF_39),[40](#_ENREF_40)] |
| P70336_AGC_DMPK | Extra Rho-bind,HR1,Myosin-tail domain and no CNH and PBD |  | Hybrid | Mouse | This domain architecture compensates for the absence of CNH and PBD domains. However, binding specificities are likely to be altered. | | [[39](#_ENREF_39),[40](#_ENREF_40)] |
| Q13464_AGC_DMPK | Extra Rho-bind,HR1,Myosin-tail domain and no CNH and PBD domains |  | Hybrid | Human | This domain architecture compensates for the absence of CNH and PBD domains. However, binding specificities are likely to be altered. | | [[39](#_ENREF_39),[40](#_ENREF_40)] |
| Q6DT37_AGC_DMPK | No PBD and PH domains |  | Hybrid | Human | PH in necessary to localize to membrane and PBD are required for binding to Rho. Due to the absence of these domains it is likely to be localised in cytosol with altered interactions. | | [[41](#_ENREF_41)] |
| Q7TT50_AGC_DMPK | No PBD and PH domains |  | Hybrid | Mouse | PH in necessary to localize to membrane and PBD are required for binding to Rho. Due to the absence of these domains it is likely to be localised in cytosol with altered interactions. | | [[41](#_ENREF_41)] |
| P92199_AGC_DMPK | No CNH,PBD and PH |  | Hybrid | Caeel | PH in necessary to localize to membrane and CNH and PBD are required for interaction with GTPases and Rho. Due to the absence of these domains it is likely to be localised in cytosol. | | [[42](#_ENREF_42)],  [[41](#_ENREF_41)] |
| Q80UW5_AGC_DMPK | Extra Filament domain |  | Hybrid | Mouse | DMPK kinase plays a role in maintenance of muscle cell. It also controls the integrity of nuclear envelope during differentiation. Filament domain helps in localization to cytoskeleton or nuclear envelope thus enabling its function. | | [[43](#_ENREF_43)] |
| Q9W1B0_AGC_DMPK | No PH domain |  | Hybrid | Drome | Likely to be localized in the cytosol. | |  |
| Q13237_AGC_PKG | Extra ATG16 | * | Rogue | Human | ATG is associated with autophagy. This protein is lost during immortalization. Entry into apoptotic pathways. | | [[44](#_ENREF_44)] |
| Q13976_AGC_PKG | Extra Med4 domain | * | Rogue | Human | Mediator between proteins and transcription machinery. Helps maintain basal transcription. | | [[45](#_ENREF_45),[46](#_ENREF_46),[47](#_ENREF_47)] |

1. Doerks T, Copley RR, Schultz J, Ponting CP, Bork P (2002) Systematic identification of novel protein domain families associated with nuclear functions. Genome research 12: 47-56.

2. Bazan JF, Fletterick RJ, Pilkis SJ (1989) Evolution of a bifunctional enzyme: 6-phosphofructo-2-kinase/fructose-2, 6-bisphosphatase. Proceedings of the National Academy of Sciences 86: 9642-9646.

3. Shoval Y, Berissi H, Kimchi A, Pietrokovski S (2011) New modularity of DAP-kinases: alternative splicing of the DRP-1 gene produces a ZIPk-like isoform. PloS one 6: e17344.

4. Michale B, Noor E, Laurie G, Emily HF, Maria D, et al. (2011) TTBK2 kinase substrate specificity and the impact of spinocerebellar-ataxia-causing mutations on expression, activity, localization and development. Biochemical Journal 437: 157-167.

5. Alzu A, Bermejo R, Begnis M, Lucca C, Piccini D, et al. (2012) Senataxin associates with replication forks to protect fork integrity across RNA-polymerase-II-transcribed genes. Cell 151: 835-846.

6. Lee S, Fan S, Makarova O, Straight S, Margolis B (2002) A novel and conserved protein-protein interaction domain of mammalian Lin-2/CASK binds and recruits SAP97 to the lateral surface of epithelia. Molecular and cellular biology 22: 1778-1791.

7. Sultana A, Kallio P, Jansson A, Wang JS, Niemi J, et al. (2004) Structure of the polyketide cyclase SnoaL reveals a novel mechanism for enzymatic aldol condensation. The EMBO journal 23: 1911-1921.

8. Hoelz A, Nairn AC, Kuriyan J (2003) Crystal Structure of a Tetradecameric Assembly of the Association Domain of Ca< sup> 2+</sup>/Calmodulin-Dependent Kinase II. Molecular cell 11: 1241-1251.

9. Magrane M (2011) UniProt Knowledgebase: a hub of integrated protein data. Database: The Journal of Biological Databases & Curation 2011.

10. da Silva Xavier G, Farhan H, Kim H, Caxaria S, Johnson P, et al. (2011) Per-arnt-sim (PAS) domain-containing protein kinase is downregulated in human islets in type 2 diabetes and regulates glucagon secretion. Diabetologia 54: 819-827.

11. Soliz J, Soulage C, Borter E, van Patot MT, Gassmann M (2008) Ventilatory responses to acute and chronic hypoxia are altered in female but not male Paskin-deficient mice. American Journal of Physiology-Regulatory, Integrative and Comparative Physiology 295: R649-R658.

12. Borden KL, Freemont PS (1996) The RING finger domain: a recent example of a sequence—structure family. Current opinion in structural biology 6: 395-401.

13. Kim L, Wong TW (1998) Growth factor-dependent phosphorylation of the actin-binding protein cortactin is mediated by the cytoplasmic tyrosine kinase FER. Journal of Biological Chemistry 273: 23542-23548.

14. Pitulescu ME, Adams RH (2010) Eph/ephrin molecules—a hub for signaling and endocytosis. Genes & development 24: 2480-2492.

15. Roszmusz E, Patthy A, Trexler M, Patthy L (2001) Localization of disulfide bonds in the frizzled module of Ror1 receptor tyrosine kinase. Journal of Biological Chemistry 276: 18485-18490.

16. Karginov AV, Ding F, Kota P, Dokholyan NV, Hahn KM (2010) Engineered allosteric activation of kinases in living cells. Nature biotechnology 28: 743-747.

17. Hantschel O, Wiesner S, Güttler T, Mackereth CD, Rix LLR, et al. (2005) Structural basis for the cytoskeletal association of Bcr-Abl/c-Abl. Molecular cell 19: 461-473.

18. Farhan H, Wendeler MW, Mitrovic S, Fava E, Silberberg Y, et al. (2010) MAPK signaling to the early secretory pathway revealed by kinase/phosphatase functional screening. The Journal of cell biology 189: 997-1011.

19. Ueno H, Honda H, Nakamoto T, Yamagata T, Sasaki K, et al. (1997) The phosphatidylinositol 3′ kinase pathway is required for the survival signal of leukocyte tyrosine kinase. Oncogene 14.

20. Yamada S, Nomura T, Takano K, Fujita S, Miyake M, et al. (2008) Expression of a chimeric CSF1R-LTK mediates ligand-dependent neurite outgrowth. Neuroreport 19: 1733-1738.

21. Hall TMT (2005) Multiple modes of RNA recognition by zinc finger proteins. Current opinion in structural biology 15: 367-373.

22. Ferrante AW, Reinke R, Stanley ER (1995) Shark, a Src homology 2, ankyrin repeat, tyrosine kinase, is expressed on the apical surfaces of ectodermal epithelia. Proceedings of the National Academy of Sciences 92: 1911-1915.

23. Ranganathan R, Ross EM (1997) PDZ domain proteins: scaffolds for signaling complexes. Current biology 7: R770-R773.

24. Sica GL, Zhu G, Tamada K, Liu D, Ni J, et al. (2001) RELT, a new member of the tumor necrosis factor receptor superfamily, is selectively expressed in hematopoietic tissues and activates transcription factor NF-κB. Blood 97: 2702-2707.

25. BRADLEY BA, WAGNER JJ, QUARMBY LM (2004) Identification and Sequence Analysis of Six New Members of the NIMA‐related Kinase Family in Chlamydomonas. Journal of Eukaryotic Microbiology 51: 66-72.

26. Thanos CD, Goodwill KE, Bowie JU (1999) Oligomeric structure of the human EphB2 receptor SAM domain. Science 283: 833-836.

27. Ohki S-y, Eto M, Kariya E, Hayano T, Hayashi Y, et al. (2001) Solution NMR structure of the myosin phosphatase inhibitor protein CPI-17 shows phosphorylation-induced conformational changes responsible for activation. Journal of molecular biology 314: 839-849.

28. Schultz J, Bork P, Ponting CP, Hofmann K (1997) SAM as a protein interaction domain involved in developmental regulation. Protein Science 6: 249-253.

29. Hollenbach AD, McPherson CJ, Mientjes EJ, Iyengar R, Grosveld G (2002) Daxx and histone deacetylase II associate with chromatin through an interaction with core histones and the chromatin-associated protein Dek. Journal of Cell Science 115: 3319-3330.

30. Mazerik JN, Tyska MJ (2012) Myosin-1a targets to microvilli using multiple membrane binding motifs in the tail homology 1 (TH1) domain. Journal of Biological Chemistry 287: 13104-13115.

31. Slaughter BD, Huff JM, Wiegraebe W, Schwartz JW, Li R (2008) SAM domain-based protein oligomerization observed by live-cell fluorescence fluctuation spectroscopy. PloS one 3: e1931.

32. Sasagawa Y, Urano T, Kohara Y, Takahashi H, Higashitani A (2003) Caenorhabditis elegans RBX1 is essential for meiosis, mitotic chromosomal condensation and segregation, and cytokinesis. Genes to Cells 8: 857-872.

33. Klug A (1999) Zinc finger peptides for the regulation of gene expression. Journal of molecular biology 293: 215-218.

34. Pearce LR, Komander D, Alessi DR (2010) The nuts and bolts of AGC protein kinases. Nature Reviews Molecular Cell Biology 11: 9-22.

35. Roelants FM, Baltz AG, Trott AE, Fereres S, Thorner J (2010) A protein kinase network regulates the function of aminophospholipid flippases. Proceedings of the National Academy of Sciences 107: 34-39.

36. Hofmann K, Falquet L (2001) A ubiquitin-interacting motif conserved in components of the proteasomal and lysosomal protein degradation systems. Trends in biochemical sciences 26: 347-350.

37. Hergovich A, Stegert MR, Schmitz D, Hemmings BA (2006) NDR kinases regulate essential cell processes from yeast to humans. Nature Reviews Molecular Cell Biology 7: 253-264.

38. Nielsen PR, Ellgaard L, Etzerodt M, Thogersen HC, Poulsen FM (1997) The solution structure of the N-terminal domain of alpha2-macroglobulin receptor-associated protein. Proceedings of the National Academy of Sciences of the United States of America 94: 7521-7525.

39. Shibata H, Mukai H, Inagaki Y, Homma Y, Kimura K, et al. (1996) Characterization of the interaction between RhoA and the amino-terminal region of PKN. FEBS letters 385: 221-224.

40. Flynn P, Mellor H, Palmer R, Panayotou G, Parker PJ (1998) Multiple interactions of PRK1 with RhoA. Functional assignment of the Hr1 repeat motif. The Journal of biological chemistry 273: 2698-2705.

41. Osada S, Izawa M, Koyama T, Hirai S, Ohno S (1997) A domain containing the Cdc42/Rac interactive binding (CRIB) region of p65PAK inhibits transcriptional activation and cell transformation mediated by the Ras-Rac pathway. FEBS letters 404: 227-233.

42. Chen XQ, Tan I, Leung T, Lim L (1999) The myotonic dystrophy kinase-related Cdc42-binding kinase is involved in the regulation of neurite outgrowth in PC12 cells. The Journal of biological chemistry 274: 19901-19905.

43. Bush EW, Helmke SM, Birnbaum RA, Perryman MB (2000) Myotonic dystrophy protein kinase domains mediate localization, oligomerization, novel catalytic activity, and autoinhibition. Biochemistry 39: 8480-8490.

44. Fujii M, Ogata T, Takahashi E, Yamada K, Nakabayashi K, et al. (1995) Expression of the human cGMP-dependent protein kinase II gene is lost upon introduction of SV40 T antigen or immortalization in human cells. FEBS letters 375: 263-267.

45. Rachez C, Lemon BD, Suldan Z, Bromleigh V, Gamble M, et al. (1999) Ligand-dependent transcription activation by nuclear receptors requires the DRIP complex. Nature 398: 824-828.

46. Naar AM, Beaurang PA, Zhou S, Abraham S, Solomon W, et al. (1999) Composite co-activator ARC mediates chromatin-directed transcriptional activation. Nature 398: 828-832.

47. Bourbon HM, Aguilera A, Ansari AZ, Asturias FJ, Berk AJ, et al. (2004) A unified nomenclature for protein subunits of mediator complexes linking transcriptional regulators to RNA polymerase II. Molecular cell 14: 553-557.
