# Supplementary material for: Hybrid and Rogue Kinases Encoded in the Genomes of Model Eukaryotes
Source: PLoS One. 2014 Sep 25;9(9):e107956. doi: 10.1371/journal.pone.0107956 (PMC4177888; doi:10.1371/journal.pone.0107956)
Supplement: Table S3 — Clustering of the 1498 sequences using full length alignment free method. Number of sequences, entropy score and subfamily variation for each cluster are also provided. (DOCX) [file pone.0107956.s004.docx]

Table S3. Clustering of the 1498 sequences using full length alignment free method. Number of sequences, entropy score and subfamily variation for each cluster are also provided.

| Cluster | Number of sequences | Entropy | Number of subfamilies¶ | Predominant subfamilies | Subfamilies present in the cluster |
| --- | --- | --- | --- | --- | --- |
| 1 | 14 | 0 | 1 | RSK | RSK |
| 2 | 16 | 0 | 1 | DYRK | DYRK |
| 3 | 11 | 0 | 1 | CAMK2 | CAMK2 |
| 4 | 12 | 0 | 1 | SGK | SGK |
| 5 | 16 | 0 | 1 | PKC | PKC |
| 6 | 195 | 0.67 | 29 | Fer | Abl,Ack,ALK,Axl,CCK4,Csk,DDR,EGFR,Eph,FAK,Fer,FGFR,JakA,KIN16,KIN6,LISK,Lmr,Met,Musk,PDGFR,Ret,Ror,Ryk,Src,STE11,STKR,Syk,Tie,TK-Sp1 |
| 7 | 324 | 0.77 | 55 | CAMKL | AKT,ALK,CAMK1,CAMKL,CASK,CCK4,CDK,CLK,DAPK,DCAMKL,DDR,DMPK,Dual,DYRK,FAK,Fer,GRK,GSK,InsR,IRAK,LRRK,MAPKAPK,MAST,MLCK,MLK,NDR,PDK1,PIM,PKA,PKD,RAD53,RAF,RCK,RIPK,RSK,RSKL,RSKR,Sev,SRPK,STE11,STE20,STE7,STKR,Syk,Trio,TSSK,TTBK,TTBKL,VRK,Worm10,Worm6,Worm7,Worm8,Worm9, YANK |
| 8 | 58 | 0.10 | 2 | CAMKL | CAMKL,TSSK |
| 9 | 98 | 0.35 | 8 | CDK | CDK,CDKL,DYRK,GSK,MAPK,PSK,RCK,RSK |
| 10 | 5 | 0 | 1 | GRK | GRK |
| 11 | 16 | 0 | 1 | GRK | GRK |
| 12 | 21 | 0 | 1 | CDK | CDK |
| 13 | 60 | 0.50 | 11 | PKG | AKT,CAMK1,DCAMKL,MAPKAPK,MLCK,PDK1,PHK,PKA,PKC,PKG,PSK |
| 14 | 7 | 0 | 1 | CAMKL | CAMKL |
| 15 | 10 | 0 | 1 | CK2 | CK2 |
| 16 | 12 | 0 | 1 | MAPK | MAPK |
| 17 | 22 | 0 | 1 | CK1 | CK1 |
| 18 | 6 | 0 | 1 | PKC | PKC |
| 19 | 12 | 0 | 1 | STE20 | STE20 |
| 20 | 15 | 0.14 | 2 | Trio | MLCK, Trio |
| 21 | 11 | 0 | 1 | PKA | PKA |
| 22 | 18 | 0 | 1 | STE7 | STE7 |
| 23 | 11 | 0 | 1 | PKN | PKN |
| 24 | 13 | 0 | 1 | CLK | CLK |
| 25 | 12 | 0 | 1 | STE20 | STE20 |
| 26 | 6 | 0 | 1 | MAPK | MAPK |
| 27 | 12 | 0 | 1 | STE20 | STE20 |
| 28 | 16 | 0 | 1 | MAPK | MAPK |
| 29 | 23 | 0 | 1 | STKR | STKR |
| 30 | 13 | 0 | 1 | RSK | RSK |
| 31 | 9 | 0 | 1 | PKC | PKC |
| 32 | 11 | 0 | 1 | STE20 | STE20 |
| 33 | 8 | 0 | 1 | MAPKAPK | MAPKAPK |
| 34 | 8 | 0 | 1 | STE20 | STE20 |
| 35 | 5 | 0 | 1 | CDK | CDK |
| 36 | 6 | 0 | 1 | STE7 | STE7 |
| 37 | 10 | 0 | 1 | CK1 | CK1 |
| 38 | 14 | 0 | 1 | CAMK1 | CAMK1 |
| 39 | 7 | 0 | 1 | NDR | NDR |
| 40 | 16 | 0 | 1 | CDK | CDK |
| 41 | 13 | 0.15 | 2 | STE20 | STE20,STE11 |
| 42 | 16 | 0.15 | 2 | Trk | Trk, DDR |
| 43 | 11 | 0 | 1 | AKT | AKT |
| 44 | 6 | 0 | 1 | STE20 | STE20 |
| 45 | 42 | 0 | 1 | Eph | Eph |
| 46 | 11 | 0 | 1 | MAST | MAST |
| 47 | 8 | 0 | 1 | PIM | PIM |
| 48 | 24 | 0 | 1 | Src | Src |
| 49 | 6 | 0 | 1 | STKR | STKR |
| 50 | 11 | 0 | 1 | DYRK | DYRK |
| 51 | 8 | 0 | 1 | RAF | RAF |
| 52 | 8 | 0 | 1 | STE20 | STE20 |
| 53 | 13 | 0 | 1 | FGFR | FGFR |
| 54 | 16 | 0 | 1 | MLK | MLK |
| 55 | 15 | 0 | 1 | DAPK | DAPK |
| 56 | 12 | 0 | 1 | Tec | Tec |
| 57 | 27 | 0.14 | 2 | PDGFR | PDGFR, VEGFR |
| 58 | 8 | 0 | 1 | Trbl | Trbl |
| 59 | 10 | 0 | 1 | InsR | InsR |
| 60 | 6 | 0 | 1 | MAPKAPK | MAPKAPK |
| 61 | 12 | 0 | 1 | CAMKL | CAMKL |
| 62 | 8 | 0 | 1 | PIM | PIM |
| 63 | 17 | 0 | 1 | DMPK | DMPK |
| 64 | 7 | 0 | 1 | DCAMKL | DCAMKL |
| 65 | 9 | 0 | 1 | SRPK | SRPK |
| 66 | 9 | 0 | 1 | PKD | PKD |
| 67 | 6 | 0 | 1 | YANK | YANK |

**¶**"subfamily" here has been identified by considering only kinase domain.
