# Supplementary material for: Hybrid and Rogue Kinases Encoded in the Genomes of Model Eukaryotes
Source: PLoS One. 2014 Sep 25;9(9):e107956. doi: 10.1371/journal.pone.0107956 (PMC4177888; doi:10.1371/journal.pone.0107956)
Supplement: Table S4 — List of hybrid and rogue kinases from P.falciparum. (DOCX) [file pone.0107956.s005.docx]

Table S4. List of hybrid and rogue kinases from *P.falciparum*

| Sequence and subfamily¶ | No.  AAs | Domain architecture | Hybrid/Rogue | Reason |  |
| --- | --- | --- | --- | --- | --- |
| B9ZSH8_CMGC_CDKL | 630 | Kinase catalytic domain\|392\|626 | Hybrid | Overhangs/Inserts within kinase domain | |
| C0H466_CMGC_RCK | 610 | Kinase catalytic domain\|361\|606 | Hybrid | Overhangs/Inserts within kinase domain | |
| C0H4K5_CAMK_CAMK1 | 1603 | Kinase catalytic domain\|1094\|1295 | Hybrid | Overhangs/Inserts within kinase domain | |
| C0H4P3_CAMK_CAMKL | 845 | Kinase catalytic domain\|566\|840 | Hybrid | Overhangs/Inserts within kinase domain | |
| C0H4R8_CMGC_MAPK | 1457 | Kinase catalytic domain\|1219\|1453 | Hybrid | Overhangs/Inserts within kinase domain | |
| C0H4W5_CMGC_MAPK | 892 | Kinase catalytic domain\|35\|810 | Hybrid | Overhangs/Inserts within kinase domain | |
| C0H513_CMGC_CDK | 542 | Kinase catalytic domain\|294\|539 | Hybrid | Overhangs/Inserts within kinase domain | |
| C0H514_AGC_RSK | 633 | Kinase catalytic domain\|394\|630 | Hybrid | Overhangs/Inserts within kinase domain | |
| C6KSZ6_CMGC_CDK | 705 | Kinase catalytic domain\|22\|499 | Hybrid | Overhangs/Inserts within kinase domain | |
| C6KT74_TKL_MLK | 2104 | Kinase catalytic domain\|2171\|2396 | Hybrid | Overhangs/Inserts within kinase domain | |
| O77306_CMGC_SRPK | 1338 | Kinase catalytic domain\|58\|850 | Hybrid | Overhangs/Inserts within kinase domain | |
| O77385_CMGC_CDK | 1553 | Kinase catalytic domain\|881\|1530 | Hybrid | Overhangs/Inserts within kinase domain | |
| O96134_STE_STE20 | 2485 | Kinase catalytic domain\|2117\|2352 | Hybrid | Overhangs/Inserts within kinase domain | |
| O96197_TKL_LISK | 1233 | Kinase catalytic domain\|952\|1206 | Hybrid | Overhangs/Inserts within kinase domain | |
| O96226_CAMK_CAMKL | 1714 | Kinase catalytic domain\|1223\|1707 | Hybrid | Overhangs/Inserts within kinase domain | |
| Q7KQK7_CMGC_MAPK | 508 | Kinase catalytic domain\|106\|446 | Hybrid | Overhangs/Inserts within kinase domain | |
| Q8I1Q9_CMGC_CDK | 699 | Kinase catalytic domain\|354\|656 | Hybrid | Overhangs/Inserts within kinase domain | |
| Q8I1T4_CMGC_CDK | 1339 | Kinase catalytic domain\|417\|920 | Hybrid | Overhangs/Inserts within kinase domain | |
| Q8I2P9_TKL_LRRK | 1277 | Kinase catalytic domain\|916\|1262; MORN\|45\|6; MORN\|69\|85; MORN\|92\|10; SAM_2\|555\|602 | Rogue | Overhangs/Inserts within kinase domain | |
| Q8I3C3_CMGC_CDKL | 591 | Kinase catalytic domain\|355\|589 | Hybrid | Overhangs/Inserts within kinase domain | |
| Q8I3C7_CMGC_CDK | 521 | Kinase catalytic domain\|282\|517 | Hybrid | Overhangs/Inserts within kinase domain | |
| Q8I4W3_AGC_RSK | 735 | Kinase catalytic domain\|404\|660; Kinase catalytic domain_C\|681\|703 | Hybrid | RSK subfamily usually contains two tandom kinase domains | |
| Q8I534_CAMK_CAMK1 | 509 | Kinase catalytic domain\|111\|364 | Hybrid | Overhangs/Inserts within kinase domain | |
| Q8I637_STE_STE20 | 567 | Kinase catalytic domain\|311\|564 | Hybrid | Overhangs/Inserts within kinase domain | |
| Q8IBU7_CAMK_CAMK1 | 509 | Kinase catalytic domain\|240\|509 | Hybrid | Overhangs/Inserts within kinase domain | |
| Q8IDK6_TKL_MLK | 1807 | Kinase catalytic domain\|1570\|1786;SAM_1\|1311\|1359 | Hybrid | SAM1 is usually seen in Eph receptors and induces dimerization | |
| Q8IDU4_CMGC_CDK | 931 | Kinase catalytic domain\|430\|684 | Hybrid | Overhangs/Inserts within kinase domain | |
| Q8IEG4_CAMK_CAMKL | 367 | Kinase catalytic domain\|108\|361; DUF3354\|41\|85 | Rogue | Overhangs/Inserts within kinase domain | |
| Q8IFM1_CMGC_CDKL | 622 | Kinase catalytic domain\|384\|620 | Hybrid | Overhangs/Inserts within kinase domain | |
| Q8IID2_CAMK_CAMK1 | 2265 | Kinase catalytic domain\|1821\|2077; efhand\|67\|8; efhand\|101\|127 | Hybrid | Efhand is usually present in CDPK subfamily involved in Ca^2+^ binding | |
| Q8IIE7_AGC_PDK1 | 525 | Kinase catalytic domain\|36\|408 | Hybrid | Overhangs/Inserts within kinase domain | |
| Q8IIL5_CMGC_DYRK | 699 | Kinase catalytic domain\|369\|687 | Hybrid | Overhangs/Inserts within kinase domain | |
| Q8IIT5_TKL_Dicty4 | 1501 | Kinase catalytic domain\|841\|1478; SAM_1\|300\|363 | Hybrid | SAM1 is usually seen in Eph receptors and induces dimerization | |
| Q8IIV3_CAMK_CDPK | 1620 | Kinase catalytic domain\|193\|736 | Hybrid | Overhangs/Inserts within kinase domain | |
| Q8IJ21_CMGC_CDK | 913 | Kinase catalytic domain\|660\|909 | Hybrid | Overhangs/Inserts within kinase domain | |
| Q8IK91_AGC_RSK | 753 | Kinase catalytic domain\|136\|308 | Hybrid | Overhangs/Inserts within kinase domain | |
| Q8IKT6_CAMK_CAMKL | 765 | Kinase catalytic domain\|397\|647 | Hybrid | Overhangs/Inserts within kinase domain | |
| Q8IL19_CMGC_CLK | 881 | Kinase catalytic domain\|554\|874 | Hybrid | Overhangs/Inserts within kinase domain | |
| Q8IL41_CMGC_DYRK | 1268 | Kinase catalytic domain\|789\|1259 | Hybrid | Overhangs/Inserts within kinase domain | |
| Q8IL57_CAMK_CAMKL | 2247 | Kinase catalytic domain\|119\|401 | Hybrid | Overhangs/Inserts within kinase domain | |
| Q8ILF0_CMGC_MAPK | 914 | Kinase catalytic domain\|23\|312 | Hybrid | Overhangs/Inserts within kinase domain | |
| Q8ILL6_CAMK_CDPK | 284 | Kinase catalytic domain\|22\|279 | Hybrid | Overhangs/Inserts within kinase domain | |

**¶**"subfamily" here has been identified by considering only kinase domain.
