# Supplementary material for: Hybrid and Rogue Kinases Encoded in the Genomes of Model Eukaryotes
Source: PLoS One. 2014 Sep 25;9(9):e107956. doi: 10.1371/journal.pone.0107956 (PMC4177888; doi:10.1371/journal.pone.0107956)
Supplement: Text S1 — Indirect method to ascertain reliability to dendrograms generated using ClaP method. (DOCX) [file pone.0107956.s007.docx]

**Text S1: Indirect method to ascertain reliability to dendrograms generated using ClaP method**.

A set of 33 sequences from 5 different sub-families of kinases (EGFR, CAMK, CDK, PKA and STE) have been taken from SWISSPROT [[1](#_ENREF_1)]. The kinase catalytic region of these sequences have been used to generate distance matrices using two methods- CLUSTALW [[2](#_ENREF_2)]- an alignment based method and ClaP [[3](#_ENREF_3)]- an alignment free method. The distance matrices have then been used to generate dendrograms. The dendrograms are highly similar in their topology. This is illustrated as a tanglegram in Figure 1 where the order of the leaves is the same in both dendrograms. The Robinson-Foulds (RF) distance was calculated to be 6 (meaning the two trees can be inter-converted to each other by roughly performing 6 operations) as specified by HashRF software [[4](#_ENREF_4)].


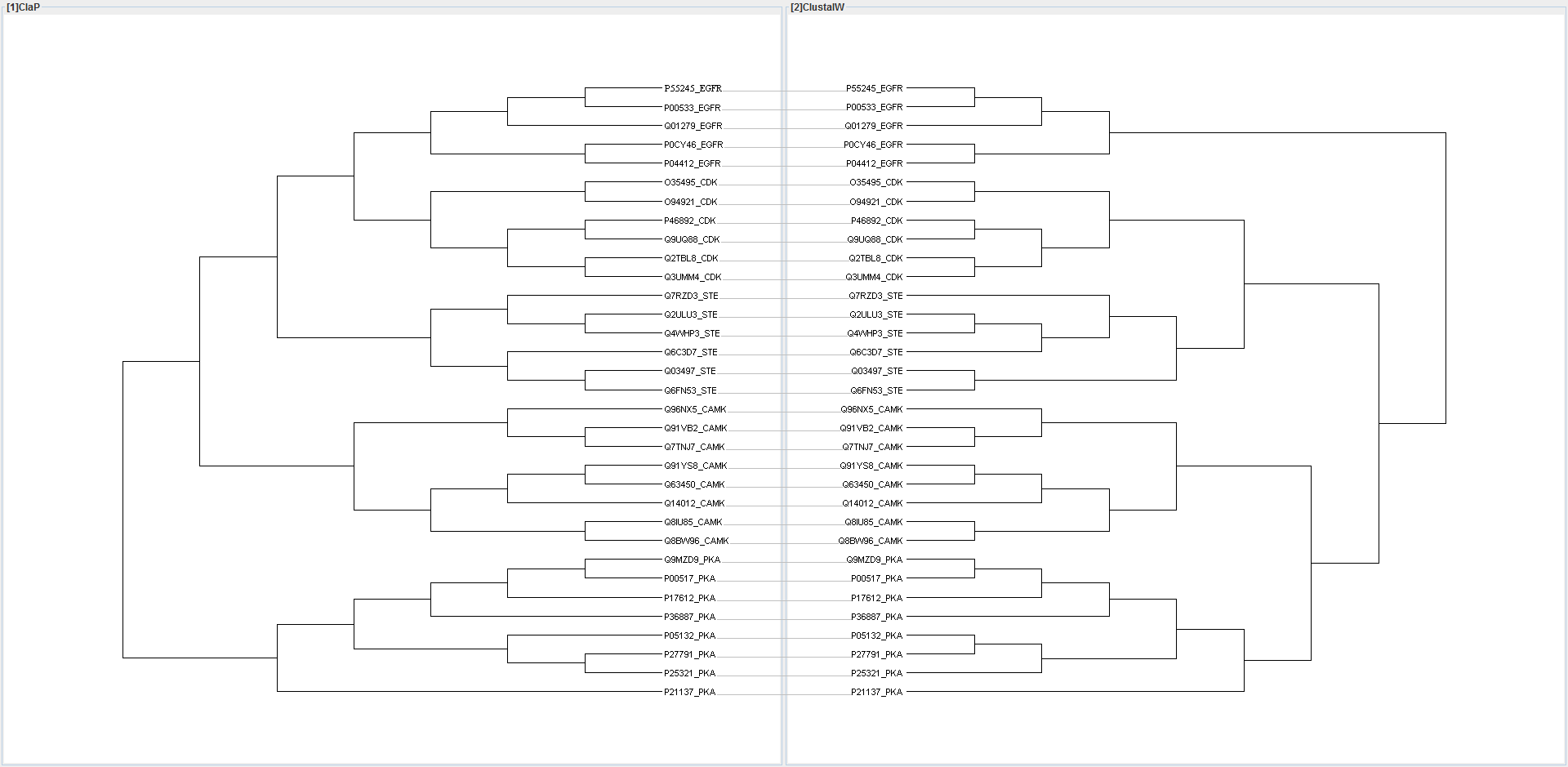


Figure 1| Tanglegram showing the similarity between the two dendrograms generated using ClaP (left) and ClustalW (right) methods. RF distance =6. Tanglegrams have been generated using Dendroscope software [[5](#_ENREF_5)].

Considering that the dendrograms are highly similar, bootstrap based reliability was performed on the ClustalW tree using MEGA5 software [[6](#_ENREF_6)] with 1000 trials and the bootstrap values are indicated in figure 2. The nodes indicate high bootstrap values (≥75 for major nodes) ascribing high reliability to the branch order of the dendrogram. This can therefore be extrapolated to the ClaP method based dendrogram.


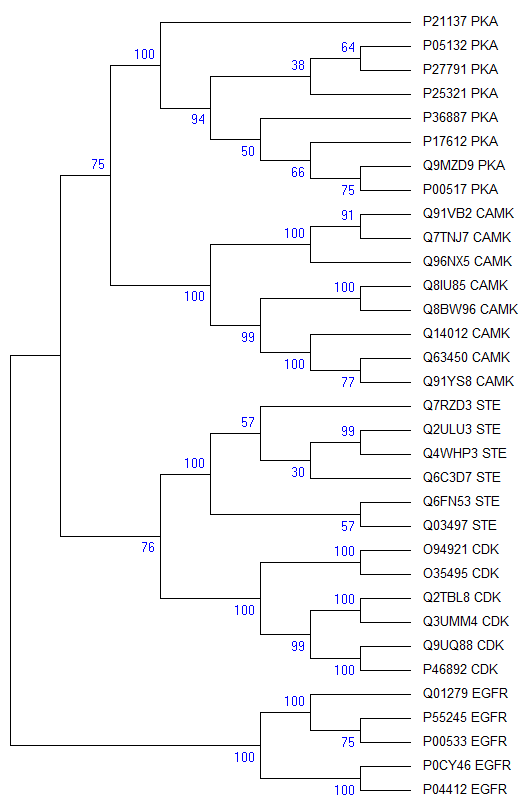


Figure 2| Bootstrap consensus dendrogram for the CLUSTALW tree using MEGA5 software.

References

1. Bairoch A, Boeckmann B, Ferro S, Gasteiger E (2004) Swiss-Prot: juggling between evolution and stability. Briefings in Bioinformatics 5: 39-55.

2. Thompson JD, Gibson T, Higgins DG (2002) Multiple sequence alignment using ClustalW and ClustalX. Current protocols in bioinformatics: 2.3. 1-2.3. 22.

3. Martin J, Anamika K, Srinivasan N (2010) Classification of protein kinases on the basis of both kinase and non-kinase regions. PloS one 5: e12460.

4. Sul S-J, Williams TL (2008) An experimental analysis of robinson-foulds distance matrix algorithms. Algorithms-ESA 2008: Springer. pp. 793-804.

5. Huson DH, Scornavacca C (2012) Dendroscope 3: an interactive tool for rooted phylogenetic trees and networks. Systematic biology: sys062.

6. Kumar S, Nei M, Dudley J, Tamura K (2008) MEGA: a biologist-centric software for evolutionary analysis of DNA and protein sequences. Briefings in Bioinformatics 9: 299-306.
